# Recurrent pattern completion drives the neocortical representation of sensory inference

**DOI:** 10.1038/s41593-025-02055-5

**Published:** 2025-09-15

**Authors:** Hyeyoung Shin, Mora B. Ogando, Lamiae Abdeladim, Uday K. Jagadisan, Severine Durand, Ben Hardcastle, Hannah Belski, Hannah Cabasco, Henry Loefler, Ahad Bawany, Josh Wilkes, Katrina Nguyen, Lucas Suarez, Tye Johnson, Warren Han, Ben Ouellette, Conor Grasso, Jackie Swapp, Vivian Ha, Ahrial Young, Shiella Caldejon, Ali Williford, Peter A. Groblewski, Shawn Olsen, Carly Kiselycznyk, Jerome Lecoq, Hillel Adesnik

**Affiliations:** 1 Department of Molecular and Cell Biology, University of California, Berkeley, Berkeley, CA USA; 2 School of Biological Sciences, Seoul National University, Seoul, Republic of Korea; 3 Allen Institute, Neural Dynamics Program, Seattle, WA USA; 4 The Helen Wills Neuroscience Institute, University of California, Berkeley, Berkeley, CA USA; 5 Department of Neuroscience, University of California, Berkeley, Berkeley, CA USA

**Keywords:** Striate cortex, Sensory processing, Extrastriate cortex, Neural decoding

## Abstract

When sensory information is incomplete, the brain relies on prior expectations to infer perceptual objects. Despite the centrality of this process to perception, the neural mechanisms of sensory inference are not understood. Here we used illusory contours (ICs), multi-Neuropixels measurements, mesoscale two-photon (2p) calcium imaging and 2p holographic optogenetics in mice to reveal the neural codes and circuits of sensory inference. We discovered a specialized subset of neurons in primary visual cortex (V1) that respond emergently to illusory bars but not to component image segments. Selective holographic photoactivation of these ‘IC-encoders’ recreated the visual representation of ICs in V1 in the absence of any visual stimulus. These data imply that neurons that encode sensory inference are specialized for receiving and locally broadcasting top-down information. More generally, pattern completion circuits in lower cortical areas may selectively reinforce activity patterns that match prior expectations, constituting an integral step in perceptual inference.

## Abstract (web summary)

Neurons that respond emergently to illusory contours drive pattern completion in V1. Pattern completion in lower cortical areas may therefore mediate perceptual inference by selectively reinforcing activity patterns that match prior expectations.

## Main

**Fig. 1:**
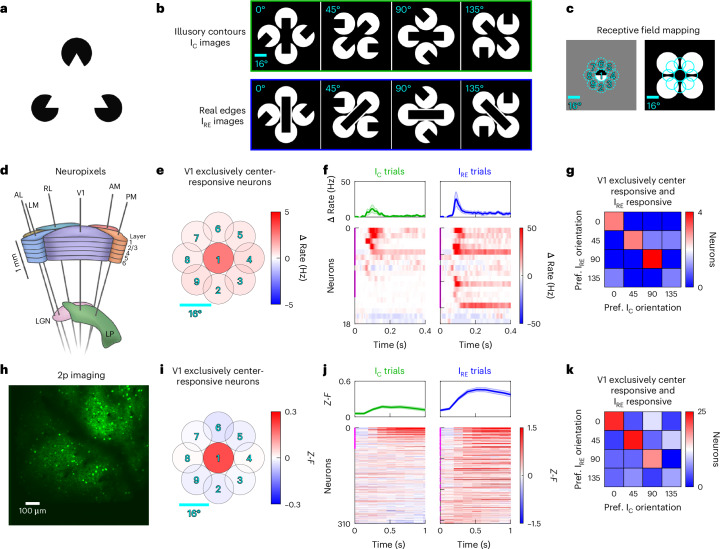
Mouse V1 neurons respond to ICs despite the lack of contrast in their receptive fields. **a**, The Kanizsa triangle illusion. **b**, Example visual stimuli used in this experiment. ‘I_C_ images’ contain illusory bars (top), while ‘I_RE_ images’ contain real bars (bottom). **c**, Receptive fields were mapped using 16° patches of circular drifting gratings appearing in one of the nine positions depicted (left). Position 1 corresponds to the gap region in the stimuli in **b** (right; for reference, the nine positions in the receptive field mapping block are shown on top of the default image from the IC block). Note that 16° is slightly larger than the typical size of receptive fields in mouse V1. **d**, Schematic of six Neuropixels probes insertion into V1, LM, RL, AL, PM and AM (12 sessions from 12 mice). **e**, Receptive field map for V1 regular-spiking (RS) units that exclusively responded to grating patches in position 1, corresponding to the illusory gap region (*n* = 18 exclusively center-responsive neurons; 12 sessions from 12 mice). **f**, PSTHs of exclusively center-responsive V1 RS units on I_C_ and I_RE_ trials, for preferred I_RE_ orientation and corresponding I_C_ trials. Top, mean ± s.e.m. across neurons. Bottom, individual neurons with substantially I_C_-responsive (left; 66.7%) and I_RE_-responsive (right; 77.8%) neurons marked in magenta on the *y* axis. **g**, Comparison of the preferred orientation between illusory bars (I_C_ stimuli) and real bars (I_RE_ stimuli) for I_RE_-responsive and exclusively center-responsive V1 RS units, showing a 78.6% match. **h**, Example 2p field-of-view of GCaMP6s expressing neurons in V1L2/3 excitatory neurons (29 sessions from five mice). **i**–**k**, Same as **e**–**g**, but for the 2p imaging dataset (*n* = 310 exclusively center-responsive V1L2/3 excitatory neurons; 29 sessions from five mice). Top, mean ± s.e.m. across exclusively center-responsive excitatory neurons in V1L2/3 (**j**). Bottom, individual neurons with substantially I_C_-responsive (left; 21.3%) and I_RE_-responsive (right; 43.2%) neurons marked in magenta on the *y* axis. There was a 54.5% match (**k**). PSTHs, peri-stimulus time histograms; LP, lateral posterior nucleus; LGN, lateral geniculate nucleus.

Illusions arise from rational mistakes in sensory inference. For example, a rational perceptual interpretation of the classic Kanizsa illusion is that of a white triangle in front of three black circles (Fig. 1a)^1^. The nervous system likely evolved to rely on inference because we must frequently infer objects from partial information, such as during occlusion^2^. Many species, including humans, nonhuman primates, mice, fish and even insects, perceive illusory contours (ICs), implying that sensory inference is fundamental to perception^3–8^. For example, mice trained to perform an orientation discrimination task on real edges can generalize their performance to ICs and vice versa^6,7^.

Illusions are uniquely suited for dissociating faithfulness from inference-based sensory representations in the brain. Pioneering work in the primate visual cortex (V1) identified neurons that responded to ICs as if they were real edges, despite the lack of any actual contrast in their receptive fields^9–11^. Such responses may represent a neural correlate of emergent, or Gestalt, perception, in which the perceived whole is greater than the sum of its parts (or sensory inputs). While lower visual cortical areas are primarily faithful to image segments, higher visual areas show stronger emergent representations of the ICs^12–14^. Furthermore, recent studies in mice showed that optogenetic silencing of a higher visual area (lateromedial area, LM) during IC presentation reduced IC-evoked responses in V1 (refs. ^7,15^). These data support a model where circuits in higher cortical areas first compute the presence of ICs and then feed back these representations as predictive inferences to lower cortical areas^16,17^. However, it remains unclear why sensory predictions are fed back to V1 at all.

To address the neocortical mechanism of sensory inference, we used large-scale neurophysiological methods to record and manipulate neural activity from awake mice viewing images that contain ICs. We used mesoscale two-photon (2p) calcium imaging, multi-Neuropixels extracellular electrophysiology recordings, 2p holographic optogenetics and a new 2p holographic mesoscope^18^. With large-scale neural recordings, we investigated how neural decoders trained to discriminate between IC stimuli generalized their performance to the discrimination of real edge stimuli. We found robust representations of IC inference in layer 2/3 (L2/3) of V1 and LM, but not in layer 4 (L4) of V1. In V1L2/3, a selected subset of neurons that responded emergently to the IC (‘IC-encoders’) mediated the representation of IC inference. To test whether these IC-encoders could drive IC representations within V1L2/3 on their own, we holographically photoactivated them in the absence of any visual stimulus. We found that photoactivating IC-encoders recreated IC representations within V1L2/3 through pattern completion. These results imply that neurons that encode top-down inference signals locally broadcast those signals through pattern completion. On the other hand, neurons that respond to the individual inducing segments of the IC (‘segment responders’) drove IC representations in higher visual areas but not within V1L2/3. Taken together, recurrent activity through feedforward, feedback and local pattern completion circuits may facilitate sensory inference by reinforcing activity patterns that match the brain’s prior expectations about the sensory world.

## Results

### Mouse V1 neurons respond to ICs despite the absence of contrast within their receptive fields

**Extended Data Fig. 1:**
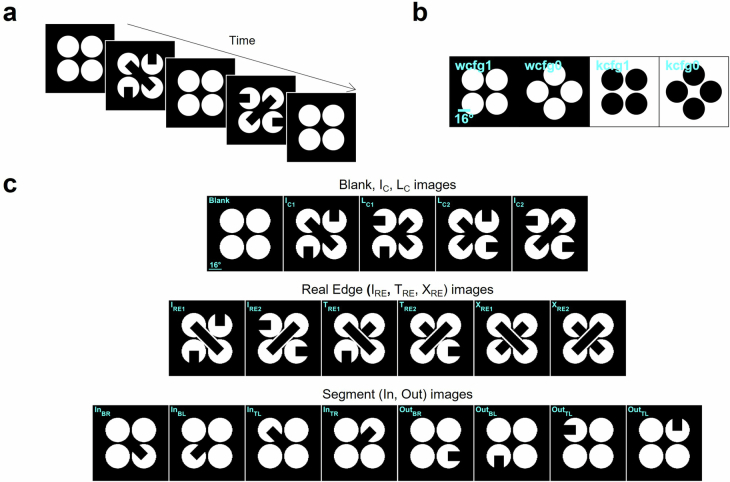
Visual stimuli used to assess neural responses to illusory contours. **a**, Schematic of visual stimulus sequences during the experiment. Throughout each IC visual block, the four circles were present in all stimulus frames. **b**, Images in the IC block were presented in four distinct types of blocks. The default block, ICwcfg1, has four white circles in each quadrant on black background. In the ICwcfg0 block, images are rotated 315° clockwise relative to ICwcfg1. ICkcfg1 and ICkcfg0 are black–white inverted from ICwcfg1 and ICwcfg0, respectively. **c**, Images shown in a ICwcfg1 block.

Using two high-throughput neural recording techniques with complementary strengths, multi-Neuropixels and large-scale 2p imaging, we recorded the neural activity of awake, head-fixed mice while presenting the animals with various images containing real edges or ICs (I_C_ denotes ‘I-shaped combination’, I_RE_ denotes ‘I-shaped real edge’; Fig. 1b and Extended Data Fig. 1).

First, we asked if we could identify visual cortical neurons that respond to ICs even in the absence of any contrast within their receptive fields, as previously shown in primates^9,10,14^. We focused on V1 neurons whose receptive fields are contained entirely within the gap region. If these neurons are driven by ICs, then their visually evoked activity is, by definition, not bottom up^7,9,10,14^. Thus, we selected only those neurons that responded exclusively to the illusory gap region, that is, the central 16 visual degree region of the visual presentation monitor (Fig. 1e,i). We then examined the response of these exclusively center-responsive V1 neurons to IC-containing images (I_C_), in comparison to nearly identical images where the ICs are replaced with real edges (‘I_RE_ images’; note that the thickness of the outline edge was ≥2°, which is detectable by mice^19^). As expected, these neurons responded more strongly to I_RE_ trials because there was actual contrast within their receptive fields. Notably, a substantial portion of these neurons also responded on I_C_ trials, despite having no contrast within their receptive fields (Fig. 1f,j). Furthermore, there was a strong degree of overlap in the orientation preference for illusory bars in I_C_ images and real bars in I_RE_ images, where the preferred I_C_ and I_RE_ orientation was defined as the orientation of the illusory bar in I_C_ images and the real bar in I_RE_ images that evoked the strongest mean response (Fig. 1g,k).

**Extended Data Fig. 2:**
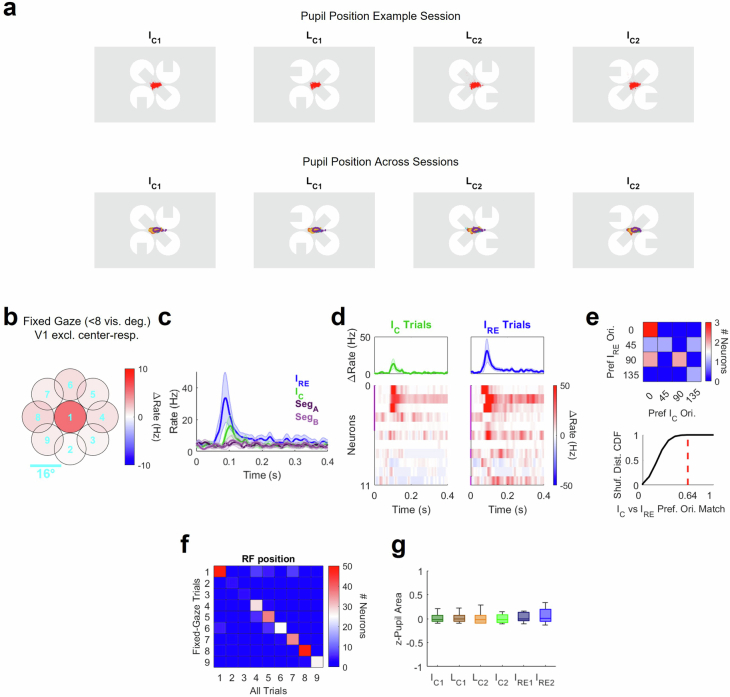
Mouse eye analysis. **a**, Top, pupil position on an example session. Bottom, contours demarcate, for each session, the isolines for the value of 0.01 in the two-dimensional probability distribution function (PDF). I_C1_ vs. L_C1_ vs. L_C2_ vs. I_C2_ images are shown in the background for relative scale. Mouse eye gaze is confined to a small region that fits within the illusory gap region. There are no systematic differences in pupil position among I_C1_ vs. L_C1_ vs. L_C2_ vs. I_C2_ presentations. **b**, Fixed-gaze trials were defined as trials where the pupil position stayed within 8 visual degrees of the mode pupil position throughout the duration of the trial. When analyzing only fixed-gaze trials, there were n = 11 exclusively center-responsive V1 RS units; 10 sessions had eye tracking data and of these, 9 sessions had a valid receptive field mapping block where the fixed-gaze constraint left at least one trial in each position. **c**, PSTH of exclusively center-responsive V1 RS units on I_C_ and I_RE_ trials, for preferred I_RE_ orientation and corresponding I_C_ and segment image trials (mean ± s.e.m. across n = 11 neurons). These neurons respond to I_RE_ images and less strongly to I_C_ images, but not to the individual segments on either side of the illusory contour. **d**, PSTH on I_C_ and I_RE_ trials for preferred I_RE_ orientation and corresponding I_C_ trials (top: mean ± s.e.m. across neurons, bottom: individual neurons with significantly I_C_-responsive (left) and I_RE_-responsive (right) neurons marked in magenta on the y axis). **e**, Top, comparison of the preferred orientation between ICs (I_C_ stimuli) and real edges (I_RE_ stimuli) for exclusively center-responsive V1 RS units. Bottom, CDF for the null distribution, acquired by shuffling the preferred I_C_ vs. I_RE_ orientations among exclusively center-responsive V1 RS units. Actual match proportion is indicated with a red dotted line. **f**, Match between receptive field position mapped with fixed-gaze trials vs. all trials. Match proportion is 0.81 among 398 V1 RS units with significant receptive fields (p < 0.05 Kruskal–Wallis test across responses to 9 positions in the receptive field mapping block). **g**, Pupil area, z-scored across each recording session, during visual presentation of various images in the IC block (n = 10 sessions with eye tracking data; p = 0.7220 Friedman test). Box-and-whisker plots are formatted as follows: centerline, median; box limits, upper and lower quartiles; whiskers, 1.5× interquartile range; circles, outliers.

**Extended Data Fig. 3:**
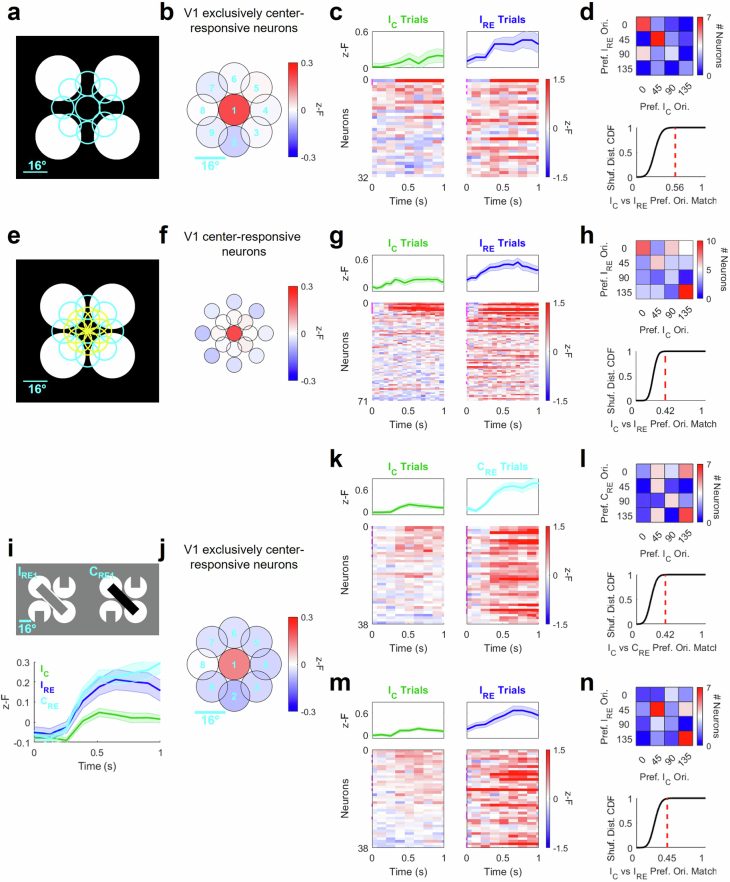
V1 layer 2/3 excitatory neurons respond to illusory contours despite the absence of contrast in their receptive fields, robustly across variations in illusory gap sizes, receptive field mapping methods and contrast vs. outline real bars. **a**, 2p imaging sessions with IC block images that had larger gaps between white circles; the gap size between diagonal white circles was 24 visual degree gaps (4 sessions from 2 mice). **b**, Receptive fields were mapped with 16 visual degree circular patches in 9 positions, spaced 16 visual degrees apart, same as Extended Data Fig. 5. There were n = 32 exclusively center-responsive V1 layer 2/3 excitatory neurons. **c**, PSTH on I_C_ and I_RE_ trials for preferred I_RE_ orientation and corresponding I_C_ trials (top: mean ± s.e.m. across neurons, bottom: individual neurons individual neurons with significantly I_C_-responsive (left) and I_RE_-responsive (right) neurons marked in magenta on the y axis). **d**, Top, comparison of the preferred orientation between ICs (I_C_ stimuli) and real edges (I_RE_ stimuli) for exclusively center-responsive V1 layer 2/3 excitatory neurons. Bottom, CDF for match proportions in shuffled distributions is shown as a black solid curve, actual match proportion in red dotted line. **e**, 2p imaging sessions with dense receptive field mapping and standard IC block images (30 visual degree white circles and 16 visual degree gaps; 5 sessions from 2 mice). **f**, Receptive fields were mapped with 16 visual degree circular patches in 17 positions, spaced 8 visual degrees apart (dense mapping, as in Extended Data Fig. 2). Of note, evoked response heatmaps are not drawn to proportion (heatmap circles have half the diameter of the actual circular patches used for mapping). Receptive field positions were determined for neurons with p < 0.05 Kruskal–Wallis test for responses across circular patch positions. There were n = 71 V1 layer 2/3 excitatory neurons with receptive fields in the center position. **g**,**h**, Same as **c** and **d**, but for V1 layer 2/3 excitatory neurons with receptive fields in the center position in dense mapping sessions. **i**, 2p imaging sessions with IC blocks that had gray background (50% contrast), with 30 visual degree white circles and 16 visual degree gaps (7 sessions from 3 mice). Top, these blocks contained contrast real edge images (C_RE_) in addition to outline real edge images (I_RE_). Bottom, PSTHs of exclusively center-responsive neurons show similar evoked responses to C_RE_ and I_RE_, and smaller evoked responses to I_C_ (mean ± s.e.m. across n = 38 exclusively center-responsive V1 layer 2/3 excitatory neurons). **j**, Receptive fields were mapped with 16 visual degree circular patches in 9 positions, spaced 16 visual degrees apart, same as **b**. **k**,**l**, Same as **c** and **d**, but for C_RE_ trials instead of I_RE_ trials in 50% contrast sessions. **m**,**n**, Same as **c** and **d**, but for 50% contrast sessions.

These results demonstrate that mouse V1 contains neurons that respond to illusory bars as if they were real bars, in an orientation selective manner, despite the lack of contrast in their receptive field. These results were consistent between the Neuropixels dataset and the 2p calcium imaging dataset. Moreover, these results did not change when limiting the analyses to trials where the mouse’s eye position stayed within eight visual degrees of the modal eye position, indicating that eye movements did not affect this conclusion (Extended Data Fig. 2). We further verified the illusory bar responsiveness and orientation selectivity for exclusively center-responsive V1 neurons using larger illusory gap sizes (Extended Data Fig. 3a–d), denser receptive field mappings (Extended Data Fig. 3e–h) and contrast bars instead of outline bars for determining orientation selectivity (Extended Data Fig. 3i–n). Taken together, V1 neurons with receptive fields within the illusory gap region can respond to illusory bars with the same orientation selectivity as to real bars.

### A subpopulation of V1 neurons respond to ICs but not to its inducing segments

Although the classical definition of IC encoding neurons are neurons whose receptive field is contained within the gap region of the IC^7,9,10,14^, neurons with receptive fields that overlap with one of the inducer segments could still be sensitive to the presence of the IC. If this were true, these neurons would be sensitive to the global arrangement of inducer elements that are outside their receptive fields. Indeed, several recent studies show that mouse V1 neurons are highly sensitive to contextual information outside their classical receptive fields, suggesting that neurons that are exclusively responsive to visual information within their classical receptive field may be more of an exception than the norm^20–23^. Thus, we sought to identify neurons that selectively encode ICs as a global emergent feature of the image, irrespective of their receptive fields.

**Fig. 2:**
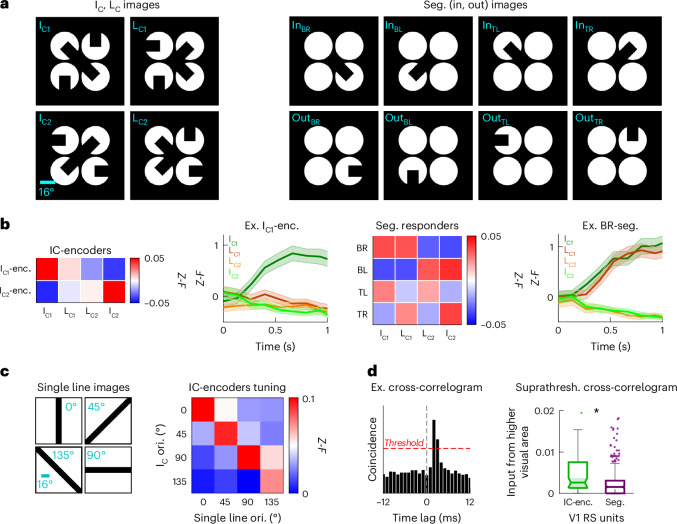
V1 IC-encoders encode global emergent features of the illusory bar. **a**, I_C1_ and I_C2_ images, respectively, contain a 135° and 45° illusory bar. L_C1_ image is constructed from bottom half of I_C1_ and top half of I_C2_, and L_C2_ is constructed from top half of I_C1_ and bottom half of I_C2_. Segment images contain individual segments of the I_C_ and L_C_ images (BR, BL, TL and TR). **b**, IC-encoders are defined as neurons that respond to one of the I_C_ images and to neither of the L_C_ images (left; two panels). Segment responders are defined as neurons that respond more to the inward than to the outward segment in each position (right; two panels). Average evoked responses of IC-encoders and segment responders to I_C_ and L_C_ images are shown as heatmaps (2p imaging dataset with V1L2/3 field-of-view, 24 sessions from four mice). Calcium traces of an example I_C1_-encoder and an example BR-segment responder neuron are shown (mean ± s.e.m. across corresponding image presentation trials). **c**, Orientation tuning of IC-encoders, measured with single long bars (left), averaged over each IC-encoder subgroup (*P* = 1.8 × 10^−5^ Wilcoxon signed-rank test between the orientation of the illusory bar vs. the orthogonal orientation; 2p imaging dataset with V1L2/3 field-of-view, 11 sessions from five mice). Orientation increases in the clockwise direction, and 0° is defined as the vertical orientation. *Y* axis denotes the orientation of the illusory bar in the I_C_ image that the IC-encoder is selective for. Of note, 135° and 45° I_C_ images correspond to I_C1_ and I_C2_ images, respectively, and 90° and 0° I_C_ images were shown in a separate block within the same recording session. **d**, Putative excitatory connections are determined as suprathreshold peaks in the cross-correlograms between RS units (example on the left). V1 IC-encoders (*n* = 79) are substantially more likely than V1 segment responders (*n* = 526) to receive putative excitatory connections from higher visual areas (LM, RL, AL, PM and AM). All box-and-whisker plots are formatted as follows: centerline, median; box limits, upper and lower quartiles; whiskers, 1.5× interquartile range; points, outliers (Wilcoxon rank-sum test *P* = 7.0 × 10^−6^; 12 sessions from 12 mice). Ex., example; enc., encoder; seg., segment; suprathresh., suprathreshold; ori., orientation.

To test this notion, we needed to design a set of visual stimuli that would distinguish between cells that simply responded to low-level features of the image per se (that is, one of the inducer segments), as opposed to the emergent IC inference. It is essential to rule out low-level image differences when identifying IC encoding neurons, such that the neural representation can be interpreted appropriately as representing sensory inference. To address this challenge, we devised a new set of four stimuli that could disambiguate low-level from emergent responses (Fig. 2a). Two of the stimuli contained illusory bars (I_C1_, I_C2_), while the other two were constructed by recombining the upper and lower halves of the first two images, such that the illusory bars were eliminated (‘L-shaped combination’, L_C1_, L_C2_). Thus, L_C1_ is composed of the bottom two inducers of I_C1_ and the top two inducers of I_C2_, and vice versa for L_C2_. This design ensured that response selectivity for one of the four images could not be explained by sensitivity to the component inducer segments; instead, it can only be explained by sensitivity to a global feature of the image. Using these stimuli, we identified approximately 4.3% of neurons in V1L2/3 with selectivity for one of the two I_C_ images, and no substantial responses to either L_C_ image (*P* < 0.05 Kruskal–Wallis test followed by Tukey–Kramer correction for multiple comparisons; 8.5% for both configurations of white-circles-on-black-background, that is, four IC orientations; Fig. 2b and Supplementary Fig. 1).

The defining feature of IC-encoders is that they respond to specific combinations of inducing segments. Conversely, neurons that respond to the individual segments per se are defined as ‘segment responders’; these neurons are substantially more responsive to inward than to outward orientation of each segment (*P* < 0.05, Wilcoxon rank-sum test; Fig. 2a). We identified segment responders in each quadrant—bottom-right (BR), bottom-left (BL), top-left (TL) and top-right (TR). Because each quadrant was shared by one of the I_C_ images and one of the L_C_ images, segment responders responded equally to an I_C_ image and an L_C_ image (Fig. 2b and Supplementary Fig. 1).

**Extended Data Fig. 4:**
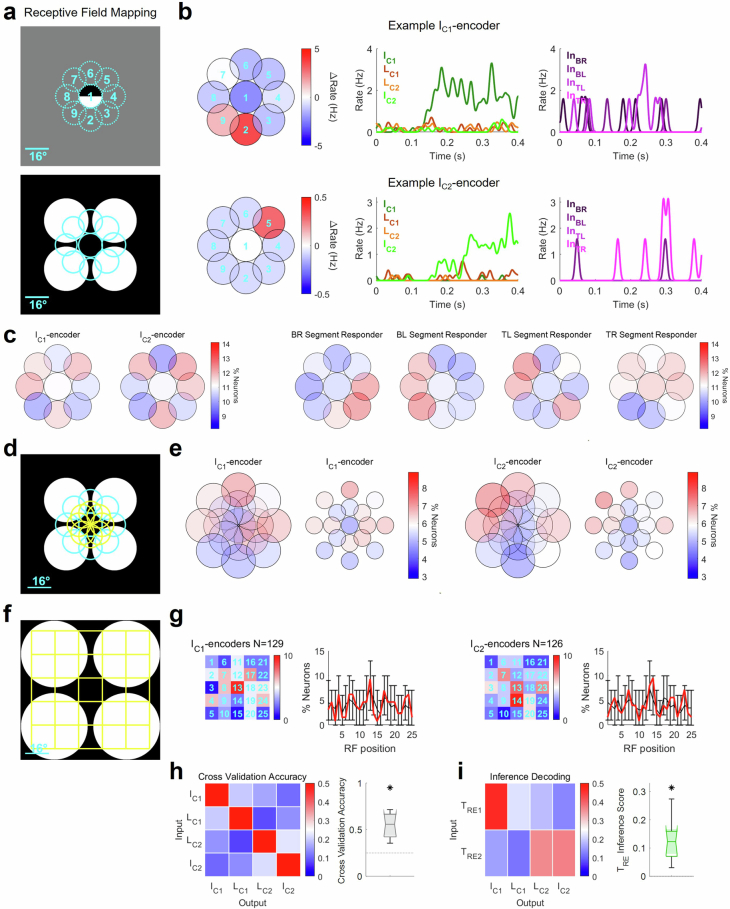
IC-encoders can have receptive fields outside of the illusory gap region. **a**, Receptive fields (RF) of all neurons were mapped using 16-degree patches of circular drifting gratings appearing in one of the nine positions depicted (top). Position 1 corresponds to the gap region in I_C_ and L_C_ images (bottom; for reference, the nine positions in the receptive field mapping block are shown on top of the default image from the IC block). Note, 16 degrees is slightly larger than the typical size of receptive fields in mouse V1. **b**, Example I_C1_-encoder (top) and I_C2_-encoder (bottom), showing its receptive field (left), PSTHs to I_C_ and L_C_ images (center), and PSTHs to inward segment images (right). PSTHs are calculated in 1 ms bins and smoothed with a Gaussian kernel that has an s.d. of 5 ms and is 25-ms long. **c**, Receptive fields percentage in each position for IC-encoders (left, two panels) and segment responders (right, four panels). IC-encoders’ receptive fields are not biased toward the illusory gap region (n = 367/386 I_C1_/I_C2_-encoders in 24 sessions from 4 mice). On the contrary, segment responders’ receptive fields are biased toward the image segments that are used to define them (n = 552/482/409/410 BR/BL/TL/TR segment responders in 24 sessions from 4 mice). **d**, Positions of circular patches used for dense receptive field mapping shown in cyan and yellow lines, relative to the IC block ‘blank’ image (19 sessions from 5 mice). **e**, IC-encoders’ receptive fields percentage in each position (n = 458/578 I_C1_/I_C2_-encoders). First and third panels show circular patches and their relative positions in actual proportions; second and fourth panels show the same data with smaller circular patches for clarity. **f**, IC block images were scaled up by 1.5× (10 sessions from 5 mice). Receptive fields were mapped with 16 visual degree square patches positioned in a 5 × 5 grid. **g**, IC-encoders’ receptive fields percentage in each position (n = 129/126 I_C1_/I_C2_-encoders). Second and fourth panels show the receptive field distribution in red, and 95% bootstrapped confidence intervals of I_C_-responsive/L_C_-responsive neurons in black. **h**–**i**, Decoder performance for images scaled up by 1.5× (n = 10 sessions from 5 mice). *p < 0.05 Wilcoxon signed-rank test compared to chance of 0.25 for cross-validation accuracy in **h** (p = 0.0020) and 0 for T_RE_ inference score in **i** (p = 0.0020). Box-and-whisker plots are formatted as follows: centerline, median; box limits, upper and lower quartiles; whiskers, 1.5x interquartile range; circles, outliers.

**Extended Data Fig. 5:**
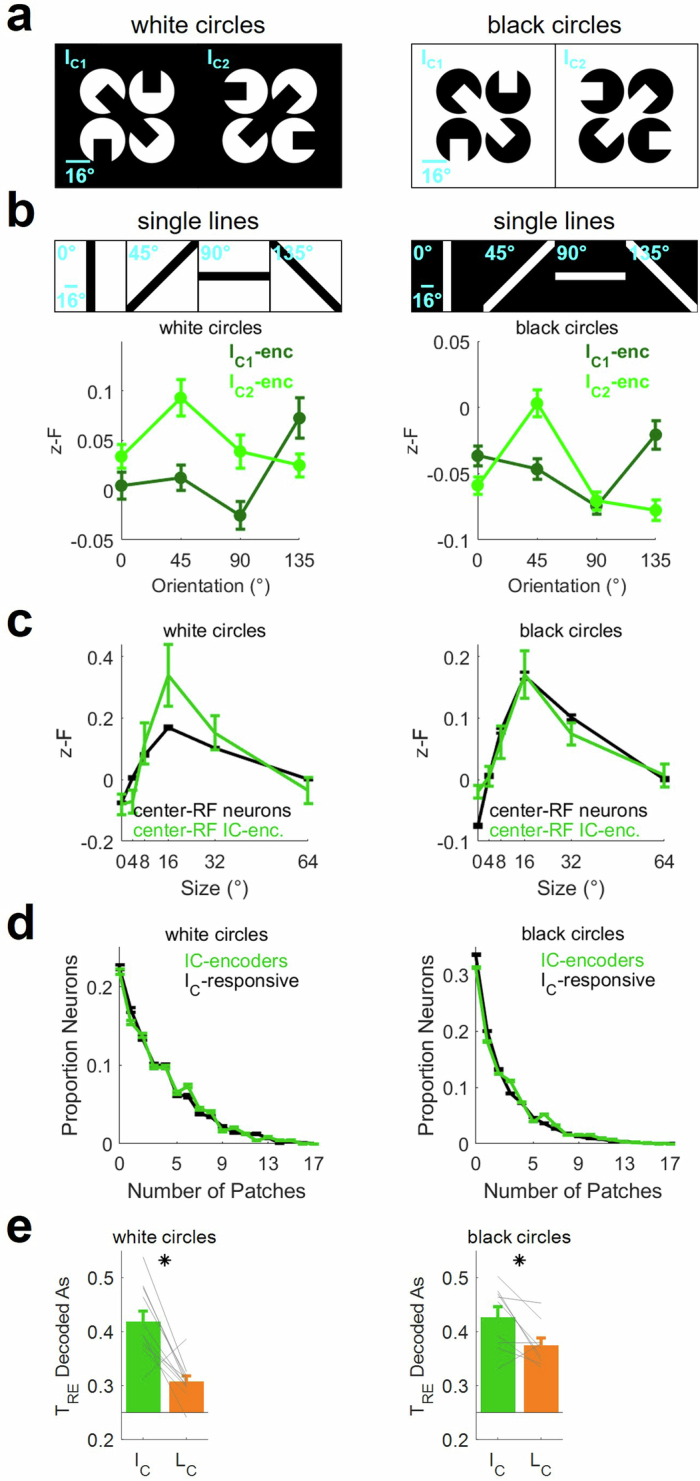
Comparison of V1 layer 2/3 IC-encoders defined in ‘white circles on black background’ blocks vs. ‘black circles on white background’ blocks. **a**, Illusory bars are perceived as dark bars on white-circles blocks (n = 11 sessions from 5 mice) and light bars on black-circles blocks (n = 9 sessions from 6 mice). **b**, IC-encoders’ orientation tuning was measured using single lines that span the entire screen; dark bars were used for IC-encoders identified in white-circles blocks and light bars for black-circles blocks. For both white-circles blocks and black-circles blocks, IC-encoders are tuned to the orientation of the illusory bar that they are selective for (mean ± s.e.m. across neurons; white-circles block n = 93 I_C1_-encoders and n = 159 I_C2_-encoders, black-circles block n = 409 I_C1_-encoders and n = 482 I_C2_-encoders; Wilcoxon signed-rank test for orientation of the illusory bar vs. orthogonal orientation p = 0.0017 in the white-circles block, p = 2.6 × 10^−8^ in the black-circles block). **c**, Size tuning was measured using circular patches of drifting gratings in various sizes, positioned at the center of the monitor. Size tuning for neurons with receptive fields in the center position is shown in black (mean ± s.e.m. across n = 1638 neurons with p < 0.05 Kruskal–Wallis test across responses to 9 positions, maximal activity in the center); size tuning for IC-encoders with receptive fields in the center is shown in green (n = 12 center-receptive field IC-encoders in the white-circles block, n = 48 in the black-circles block). Receptive field size measured in this way is not distinct for IC-encoders compared to other visually responsive neurons. **d**, Circular patches of drifting gratings with 16 visual degree diameters were shown in 17 different positions spaced 8 visual degrees apart, as shown in Extended Data Fig. 2d. Number of patches that IC-encoders respond to is not different from IC-responsive neurons (error bars indicate 95% bootstrapped confidence interval). **e**, When decoding across all neurons recorded in each V1 layer 2/3 imaging session, T_RE_-evoked neural activity patterns are more likely to be decoded as I_C_ than L_C_ on both white-circles blocks (Wilcoxon signed-rank test p = 0.0020 across n = 11 sessions) and black-circles blocks (Wilcoxon signed-rank test p = 0.0273 across n = 9 sessions). Error bars indicate mean ± s.e.m. across sessions, and gray lines indicate paired T_RE_ inference decoding performance for each session.

To test whether IC-encoders are selective to the illusory bar in the I_C_ image, we examined the orientation tuning of these neurons using single line images (Fig. 2c). Indeed, IC-encoders in V1L2/3 were tuned to the orientation of the illusory bar in the I_C_ image (I_C1_-encoders were tuned to 135°, and I_C2_-encoders were tuned to 45°). Interestingly, although some of these neurons had receptive fields that mapped to the gap region of the IC, many did not (Extended Data Fig. 4a–g). This indicates that emergent responses to the illusory bar are not constrained to neurons with receptive fields on the illusory gap region. Furthermore, IC-encoders did not have substantially larger receptive fields compared to other neurons in V1L2/3, arguing that IC-encoders’ selectivity to ICs cannot be attributed to feedforward convergence of sensory information across the illusory gap region (Extended Data Fig. 5c,d). In addition, eye movement was largely constrained within the gap region and was not biased across images, suggesting that eye movements did not influence IC-encoders’ specificity to ICs (Extended Data Fig. 2a). Together, these results imply that bottom-up visual inputs cannot fully account for IC-encoders’ responses to ICs in V1L2/3.

Top-down corticocortical feedback generates IC encoding in V1 in both primates and mice^7,14–17^. This leads to the prediction that IC-encoders receive IC inference signals through top-down feedback from higher visual areas. To test this, we leveraged the large-scale nature of the Neuropixels dataset to perform a cross-correlogram analysis as an estimate of connectivity between higher visual areas and V1 (12 sessions from 12 mice; mean ± s.e.m. = 150 ± 20 V1 RS units and 597 ± 46 higher visual area RS units across sessions). Following a prior approach, we deemed a sharp peak with a short latency in the cross-correlogram exceeding a certain threshold to be a putative excitatory connection (Fig. 2d, left; Methods)^24,25^. Under this analysis, we found that V1 IC-encoders received substantially more putative excitatory inputs from higher visual areas, compared to V1 segment responders (Fig. 2d).

### Representation of IC inference across the mouse visual cortical hierarchy

**Extended Data Fig. 6:**
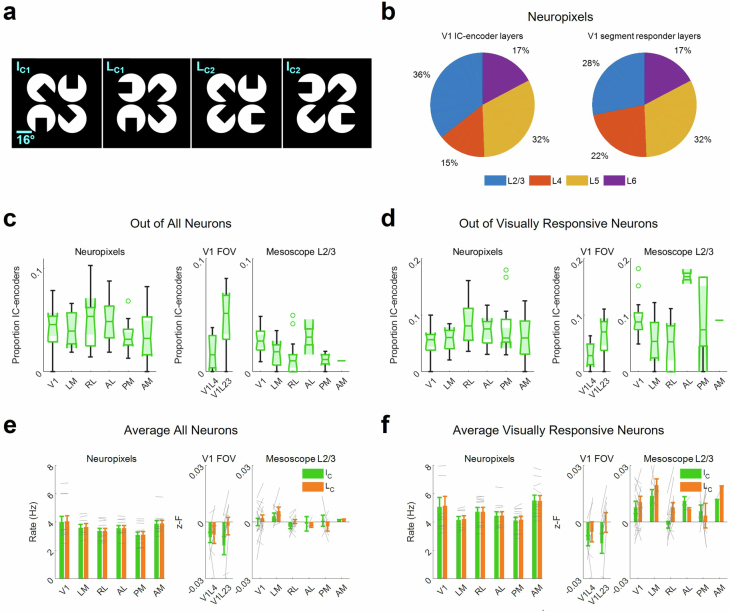
Images with illusory bars (I_C_) do not evoke greater population average responses than images without (L_C_). **a**, I_C_/L_C_ images used to define IC-encoders and segment responders in this figure. **b**, For the Neuropixels dataset, proportion of V1 IC-encoders (left, *n* = 87) and V1 segment responders (right, n = 636) in each layer. In **c**–**f**, same dataset as Fig. 3d,e (left: of 12 sessions in Neuropixels dataset, sessions with <50 RS units are excluded, which leaves n = 12, 8, 11, 10, 12 and 11 sessions for V1, LM, RL, AL, PM and AM, respectively; right: n = 8/11/19 sessions for V1L4 FOV, V1L2/3 FOV, and mesoscope FOV, respectively. For each visual area, sessions with <100 neurons in that area are excluded, which leaves n = 8, 11, 19, 8, 15, 2, 6 and 1 sessions for V1L4, V1L2/3, mesoscope V1, LM, RL, AL, PM and AM, respectively). In **c**,**d**, box-and-whisker plots are formatted as follows: centerline, median; box limits, upper and lower quartiles; whiskers, 1.5x interquartile range; circles, outliers. **c**, Proportion of IC-encoders, out of all neurons, in each area. **d**, Proportion of IC-encoders, out of visually responsive neurons, in each area. Visually responsive neurons are defined as neurons that respond to at least one of I_C_/L_C_ images (p < 0.05 Kruskal–Wallis test across responses to blank vs. I_C1_ vs. L_C1_ vs. L_C2_ vs. I_C2_). In **e**,**f**, response on I_C_ vs. L_C_ trials, averaged across all neurons (**e**) or visually responsive neurons (**f**) for each session (mean ± s.e.m. across sessions, paired data for each session indicated with gray lines). I_C_ images do not evoke greater responses than L_C_ images (*p < 0.05 one-tailed Wilcoxon signed-rank test for testing I_C_ > L_C_; note that none of the visual areas showed significance). **e**, Response on I_C_ vs. L_C_ trials, averaged across all neurons. In Neuropixels dataset, one-tailed Wilcoxon signed-rank test for testing I_C_ > L_C_ returned p = 0.9614, 0.9805, 0.7676, 0.7217, 0.9895 and 0.8398 for V1, LM, RL, AL, PM and AM, respectively. In 2p dataset, p = 0.7266, 0.9932, 0.8476, 0.8086, 0.9794, 0.5000, 0.2188 and 1.0000 for V1L4, V1L2/3, mesoscope V1, LM, RL, AL, PM and AM, respectively. **f**, Response on I_C_ vs. L_C_ trials, averaged across visually responsive neurons. In Neuropixels dataset, one-tailed Wilcoxon signed-rank test for testing I_C_ > L_C_ returned p = 0.9788, 0.9805, 0.7114, 0.7842, 0.9866 and 0.6499 for V1, LM, RL, AL, PM and AM, respectively. In 2p dataset, p = 0.8750, 0.9878, 0.8902, 0.9023, 0.9990, 0.2500, 0.3438 and 1.0000 for V1L4, V1L2/3, mesoscope V1, LM, RL, AL, PMz and AM, respectively.

To understand how IC inference is computed at the microcircuit level, we then sought to determine where in the visual hierarchy this computation occurs. In primates, this top-down information is thought to come from V2 and the ventral stream^13,14^. In mice, the functional specialization of higher visual areas is less clear, although a recent optogenetics study has shown that LM contributes to IC responses in V1 (ref. ^7^). Perhaps more notably, in both primates and mice, it is unclear whether V1 is the lowest region in the visual hierarchy where IC responses emerge. To address these questions, we recorded from six visual cortical areas simultaneously (V1, LM, anterolateral (AL), rostrolateral (RL), anteromedial (AM) and posteromedial (PM)), by targeting six Neuropixels probes to each area (*n* = 12 sessions from 12 mice), or by using a 2p mesoscope for calcium imaging in a separate cohort of mice (*n* = 19 sessions from 5 CaMKII-tTA;tetO-GcaMP6s mice; Supplementary Fig. 2). We also compared V1L2/3 (*n* = 11 sessions from five CaMKII-tTA;tetO-GcaMP6s mice) and V1L4 (*n* = 8 sessions from two Scnn1a-Tg3-Cre;TIT2L-GC6s-ICL-tTA2 mice) using multiplane imaging in a standard 2p microscope. Using the same approach as above for V1, we could identify IC-encoders throughout these visual areas both in the electrophysiology and in the calcium imaging datasets (Extended Data Fig. 6c,d).

**Fig. 3:**
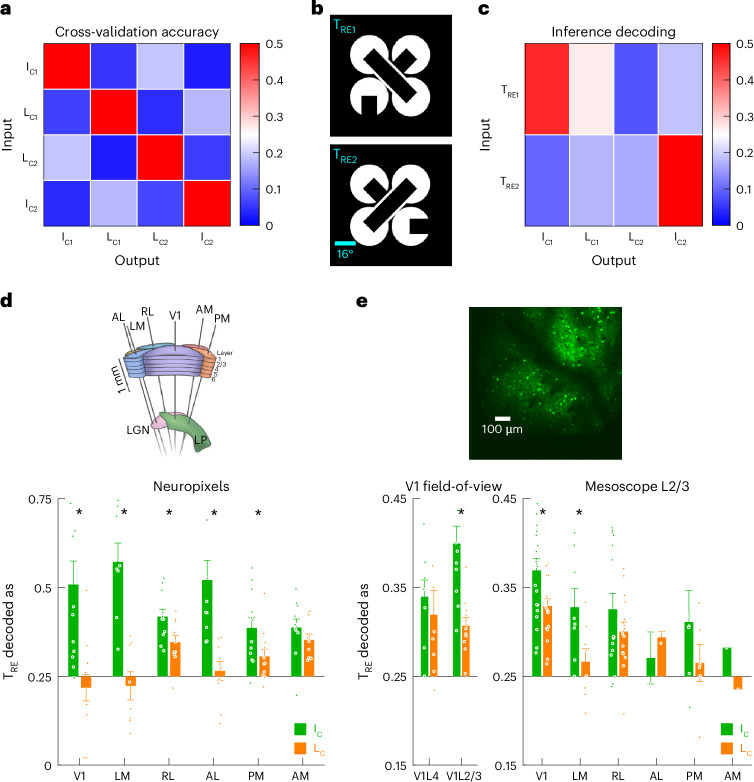
IC inference is represented in L2/3 of V1 and LM. **a**, Tenfold cross-validation performance of a linear SVM trained on neural activity evoked by I_C1_ vs. L_C1_ vs. L_C2_ vs. I_C2_ images. The confusion matrix shows the average decoding performance of V1 neurons in the Neuropixels dataset (*n* = 12 sessions from 12 mice; average 150 V1 RS units per session, 400 repetitions per trial type). **b**, The stimuli used for assessing inference decoding. T_RE_ stimuli have equivalent pixel overlap with an I_C_ and an L_C_. **c**, Inference decoding performance, obtained by probing the decoder trained on I_C_ and L_C_ trials with T_RE_ trials (same decoders as **a**). **d**, Inference decoding of each visual cortical area in the Neuropixels dataset (mean ± s.e.m. across sessions, *P < 0.05, Wilcoxon signed-rank test; for each visual area, of 12 sessions, only sessions with ≥50 RS units are included, which leaves *n* = 12, 8, 11, 10, 12, 11 sessions with *P* = 0.0132, 0.0156, 0.0244, 0.0039, 0.0342, 0.1748 for V1, LM, RL, AL, PM and AM, respectively). Green, I_C_; orange, L_C_. **e**, Inference decoding of the 2p dataset. The proportion of T_RE_ trials that are decoded as the corresponding I_C_ (green) and L_C_ (orange) images were averaged across the two image sets (mean ± s.e.m. across sessions, *P < 0.05, Wilcoxon signed-rank test; 8 sessions with V1L4 field-of-view, 11 sessions with V1L2/3 field-of-view and 19 sessions with mesoscope field-of-view; for each visual area, only sessions ≥100 neurons in that area are included, which leaves *n* = 8, 11, 19, 8, 15, 2, 6, 1 sessions with *P* = 0.3125, 0.0049, 0.0196, 0.0391, 0.2293, 1.0000, 0.4375, 1.0000 for V1L4, V1L2/3, mesoscope V1, LM, RL, AL, PM and AM, respectively).

Owing to the high-throughput nature of our recording techniques, we could apply powerful multivariate approaches that can test for the representation of IC inference comprehensively. We trained neural decoders (linear support vector machines, SVMs) to classify visual evoked neural activity patterns into corresponding visual labels for the four trial types described above (I_C1_, L_C1_, L_C2_ and I_C2_ trials; chance performance is 0.25). The tenfold cross-validation performance was substantially above chance, indicating that neural activity patterns evoked by these four images were highly distinct at the multivariate level (Fig. 3a).

To test whether IC inference is represented in the visually evoked neural activity patterns, we designed two new images to probe the neural decoder with, called ‘T_RE_ images’ (Fig. 3b). T_RE1_ has equal pixel overlap with I_C1_ and L_C1_, and, correspondingly, T_RE2_ has equal pixel overlap with I_C2_ and L_C2_. T_RE_ images contain explicit real edges, unlike any of the four stimuli used to train the decoder (I_C1_, L_C1_, L_C2_ and I_C2_). If visually evoked activity patterns were faithful to the images, decoder output would simply reflect the overlap between the probe images (T_RE1_ and T_RE2_) and the training images (I_C1_, L_C1_, L_C2_ and I_C2_). Thus, the decoder would be equally likely to classify T_RE_-evoked neural activity patterns as I_C_ or L_C_. On the contrary, if IC inference was represented in I_C_-evoked activity, the corresponding activity pattern would emulate the representation of the real edge in the T_RE_-evoked activity. Thus, if a brain area contained IC inference signals, the T_RE_-response in that area would be more similar to the I_C_-response than to the L_C_-response. To quantify this effect, we computed the difference in the classification of T_RE_ as I_C_ vs. L_C_ (averaged across the two image sets). We termed this analysis ‘inference decoding’. Applying inference decoding to Neuropixels V1 recordings revealed that T_RE_ was substantially more likely to be decoded as I_C_ than as L_C_ (Fig. 3c; decoding probability for I_C_ vs. L_C_ was 0.51 vs. 0.22, *P* < 0.05 Wilcoxon signed-rank test). This result indicates that IC inference is represented in mouse V1.

To narrow down the lowest region of the visual hierarchy where the inference signal is encoded, we used 2p imaging to examine V1L4 and L2/3 separately. Whereas V1L2/3 showed substantial inference decoding performance, V1L4 did not (Fig. 3e). This result is consistent with the notion that V1L4, as the primary thalamo-recipient layer, is more faithful to sensory information than V1L2/3. Critically, it implies that V1L2/3 is the lowest region in the visual hierarchy where IC inference is represented.

Next, we applied T_RE_ inference decoding to identify higher visual areas that participate in IC encoding (Fig. 3d,e; *P < 0.05 Wilcoxon signed-rank test). In the Neuropixels dataset, almost every visual area was substantially biased toward decoding T_RE_ as I_C_ than L_C_, with V1, LM and AL showing the strongest representations of the IC (Fig. 3d). Similarly, L2/3 of V1 and LM showed the strongest IC representation in the 2p mesoscope dataset (Fig. 3e). The difference in effect sizes between the 2p imaging and the Neuropixels data likely stems from inherent differences in signal-to-noise ratio between calcium imaging and extracellular electrophysiology.

**Extended Data Fig. 7:**
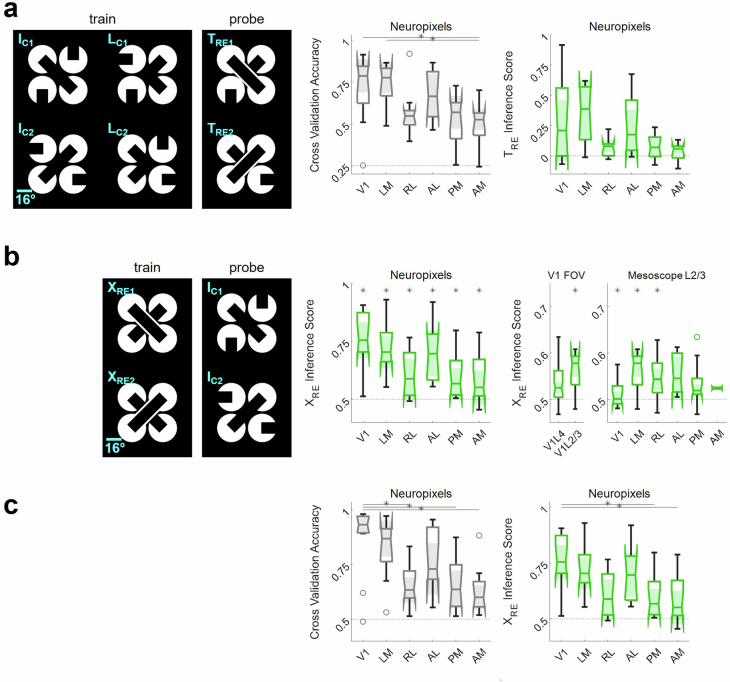
Inference decoding based on X_RE_ images shows consistent results with T_RE_-based inference decoding. In **a**–**c**, box-and-whisker plots are formatted as follows: centerline, median; box limits, upper and lower quartiles; whiskers, 1.5x interquartile range; circles, outliers. **a**, Same data as Fig. 2, but showing statistical comparisons between areas (12 sessions from 12 mice; for each area in the Neuropixels dataset, sessions with <50 RS units are excluded, which leaves n = 12, 8, 11, 10, 12 and 11 sessions for V1, LM, RL, AL, PM and AM, respectively; Kruskal–Wallis test p = 0.0019 for cross-validation accuracy across areas, p = 0.0249 for T_RE_ inference score). **b**, X_RE_ inference decoding entails probing I_C_-evoked neural activity patterns with decoders trained to discriminate between X_RE_-evoked neural activity patterns. X_RE_ inference score was defined as (P(I_C1_ → X_RE1_) + P(I_C2_ → X_RE2_))/2. As with T_RE_ inference decoding (Fig. 2), layer 2/3 of V1 and LM consistently show high inference scores (*p < 0.05, Wilcoxon signed-rank test compared to chance of 0.5; left: of 12 sessions in Neuropixels dataset, sessions with <50 RS units are excluded, which leaves n = 12, 8, 11, 10, 12 and 11 sessions with p = 0.0005, 0.0078, 0.0049, 0.0020, 0.0005 and 0.0137 for V1, LM, RL, AL, PM and AM, respectively; right: n = 8/11/19 sessions for V1L4 FOV, V1L2/3 FOV, and mesoscope FOV, respectively. For each visual area, sessions with <100 neurons in that area are excluded, which leaves n = 8, 11, 19, 8, 15, 2, 6 and 1 sessions with p = 0.4609, 0.0020, 0.0007, 0.0078, 0.0054, 0.5000, 0.4375 and 1.0000 for V1L4, V1L2/3, mesoscope V1, LM, RL, AL, PM, and AM, respectively). **c**, Same data as **b**, but showing statistical comparisons between areas (Kruskal–Wallis test p = 0.0005 for cross-validation accuracy across areas, p = 0.0022 for X_RE_ inference score).

Prior behavioral work has shown that mice trained on real edges could generalize to ICs^6^, and, conversely, mice trained on ICs could generalize to real edges^7^. Analogously, we developed a complementary inference decoding approach where the neural decoder was trained on real edges and then probed with ICs. To this end, we designed a new set of images that have equal pixel overlap with I_C1_ and I_C2_ images; referred to as ‘X_RE_ images’ (Extended Data Fig. 7b). The X_RE1_ and X_RE2_ images each have a real bar in the foreground, at the same position and orientation as the illusory bar in the I_C1_ and I_C2_ images, respectively. If neural responses are faithful to sensory inputs, we would expect both I_C1_ and I_C2_ to be equally likely to be decoded as X_RE1_ and X_RE2_. Alternatively, if the illusory bars in the I_C_ images are represented in the neural activity pattern, the I_C_-evoked activity would be decoded as the X_RE_ image with the real bar that matches the illusory bar. We trained the neural decoders to discriminate between neural activity patterns evoked by X_RE1_ vs. X_RE2_ images and used those decoders to classify I_C1_-evoked and I_C2_-evoked neural activity patterns. Then we computed the X_RE_ inference score as the average of the probability that I_C1_ was decoded as X_RE1_, and I_C2_ as X_RE2_, and tested whether the X_RE_ inference score was substantially different from chance of 0.5. When applying X_RE_ inference decoding to different visual areas, we found consistent results as T_RE_ inference decoding—IC inference was most strongly represented in L2/3 of V1 and LM (Extended Data Fig. 7b).

As a further test of the robustness of inference decoding, we applied this analysis to black-and-white inverted images. We found that inference decoding indicated the representation of IC inference in V1L2/3 regardless of black–white inversion (Extended Data Fig. 5e). In addition, different types of decoders yielded highly consistent results (Supplementary Fig. 3). These results indicate that inference decoding is a robust indicator of IC inference.

Together, our data support a model in which the neocortical representation of IC inference arises through corticocortical feedforward and feedback pathways between V1 and higher visual areas, especially LM. Furthermore, IC inference is encoded in V1L2/3 and not V1L4, indicating that V1L2/3 is the lowest region in the visual hierarchy where IC inference signals emerge. Notably, none of the higher visual areas showed a substantially larger inference performance compared to V1 (Extended Data Fig. 7a,c). This suggests that V1L2/3 may have an active role in shaping IC inference, rather than passively reflecting the inference signals computed in higher visual areas. Thus, we sought to elucidate the role of V1L2/3 in IC encoding.

### Neurons that respond emergently to ICs mediate the representation of inference in V1

So far, our data have established mechanisms of IC encoding at two levels. At the univariate level, we identified IC-encoders as neurons that respond to the whole illusory bar, but not its individual segments (Fig. 2). At the multivariate level, we found the representation of IC inference in the neural activity patterns (Fig. 3). To connect these two levels, we sought to assess the contribution of IC-encoders to the multivariate representation of IC inference. We focused on V1L2/3 because it is the lowest region in the visual hierarchy where IC inference is represented. We used the 2p imaging data for this analysis since it afforded the necessary dense sampling of V1L2/3. Moreover, we wanted to eventually test the causal role of functionally identified neural ensembles, and 2p imaging is readily compatible with 2p holographic optogenetics experiments.

**Fig. 4:**
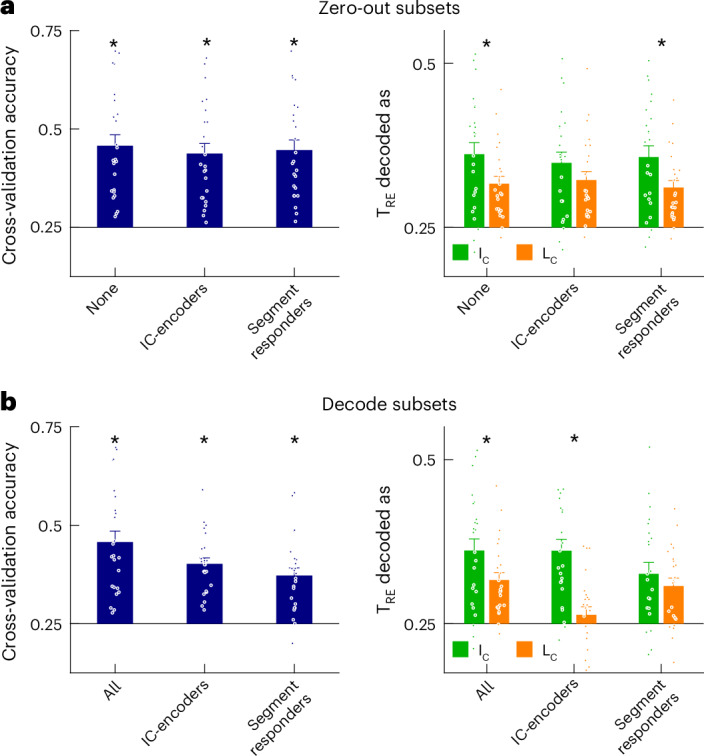
IC-encoders mediate the representation of IC inference in V1L2/3. **a**, Decoder prediction performance when zeroing out the indicated subsets of neurons in the decoder input after training the decoder (mean ± s.e.m. across *n* = 24 sessions from four mice; left—cross-validation accuracy Friedman test across subsets *P* = 1.1 × 10^−5^, *P < 0.05 Wilcoxon signed-rank test compared to chance performance of 0.25 with *P* = 1.8 × 10^−5^, 1.8 × 10^−5^, 1.8 × 10^−5^ for each subset; right—T_RE_ inference performance Friedman test across subsets *P* = 0.0019, *P < 0.05 Wilcoxon signed-rank test comparing fraction of I_C_ vs. L_C_ trials decoded as the corresponding T_RE_ with *P* = 0.0177, 0.1228, 0.0089 for each subset). **b**, Decoder performance when decoding only from the indicated subsets of neurons (same subsets as **a**; cross-validation accuracy Friedman test *P* = 0.0025 and Wilcoxon signed-rank test *P* = 1.8 × 10^−5^, 1.8 × 10^−5^, 5.6 × 10^−5^; T_RE_ inference score Friedman test *P* = 0.0208 and Wilcoxon signed-rank test *P* = 0.0177, 0.0004, 0.2472; mean ± s.e.m. across *n* = 24 sessions from four mice, *P < 0.05 Wilcoxon signed-rank test).

To test whether IC-encoders are essential for the multivariate representation of IC inference, we analyzed the change in decoder performance when zeroing out their activity in the input to the decoder (Fig. 4a; V1L2/3 field of view, 24 sessions from four mice; mean ± s.e.m. = 1,968 ± 131 neurons per session). Indeed, this abolished the IC inference performance; T_RE_-responses were no longer biased toward being classified as I_C_ compared to L_C_ (Fig. 4a, right). IC-encoders are defined only by their selective visual responses to I_C_ compared to L_C_, independent of any analysis of decoder weights. Thus, zeroing them out in the decoder can be meaningfully interpreted as being essential for decoding.

Next, we took the converse approach and trained a new decoder only on IC-encoders (Fig. 4b; mean ± s.e.m. = 30 ± 4 IC-encoders across sessions). Despite training this decoder on the activity of drastically fewer cells, it showed high levels of IC inference performance (Fig. 4b, right). These results demonstrate that the neural representation of IC inference is almost entirely mediated by the activity of IC-encoders.

IC encoding is first initiated by bottom-up sensory drive from the inducing segments of the IC^16,17^. Thus, neurons that respond to the inducing segments, that is, segment responders, likely have an important role in feedforward sensory processing. As expected, segment responders contained sufficient visual information to discriminate between I_C_ and L_C_ images on their own (Fig. 4b, left). However, in contrast to IC-encoders, zeroing out segment responders’ activity in the original decoder did not interfere with IC inference performance (Fig. 4a, right), and training a new decoder only on these neurons did not yield a substantial IC inference performance (Fig. 4b, right; mean ± s.e.m. = 69 ± 8 segment responders across sessions). These results suggest that segment responders faithfully encode sensory information contained in the image, rather than global emergent features of the image. Furthermore, these results implicate a functional dichotomy between segment responders and IC-encoders, where the former receive bottom-up sensory signals and the latter receive top-down inference signals (Supplementary Fig. 4).

Taken together, we found that IC-encoders are disproportionately important for the neural representation of IC inference in V1L2/3. Furthermore, these results imply a segregation of inputs; segment responders receive bottom-up sensory signals and IC-encoders receive top-down inference signals. Next, we investigated the output connectivity of these V1L2/3 ensembles using 2p holographic optogenetics.

### Neurons that encode IC inference drive neural pattern completion within V1

Prior studies have shown that IC encoding in V1 arises through top-down feedback^7,15–17^. However, prior work does not explain why IC encoding should exist in V1 in the first place, nor whether it might actively contribute to IC encoding. To address these questions, we used 2p holographic optogenetics to examine the direct causal role of distinct V1L2/3 ensembles in shaping IC encoding.

**Fig. 5:**
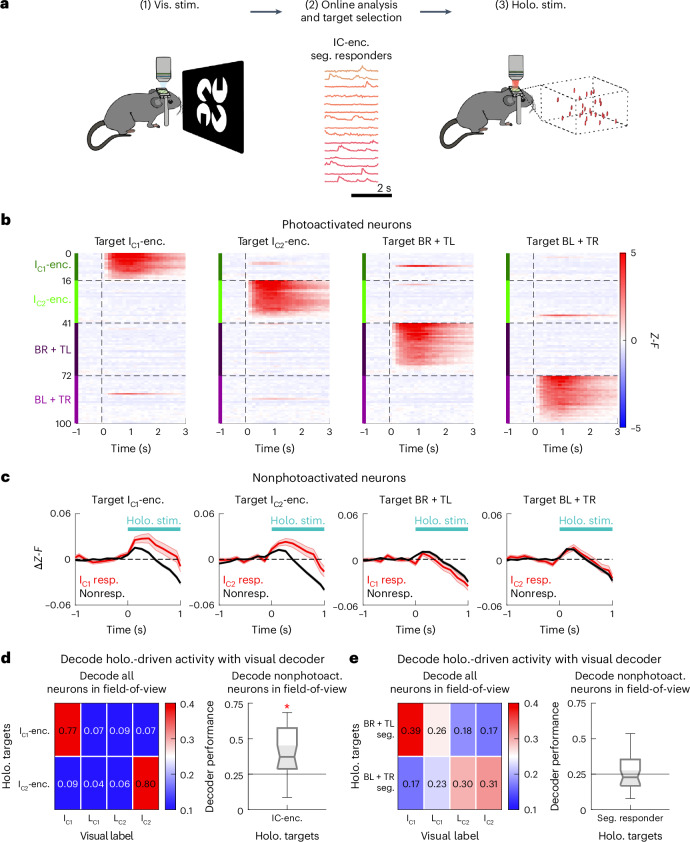
The 2p holographic optogenetic stimulation of IC-encoders drives recurrent pattern completion of IC representations in V1L2/3. **a**, ‘All-optical read-write’ experimental pipeline (24 sessions from four mice expressing GCaMP6m–ChRmine in V1 neurons). In stage 1 (‘read’ stage), each image was presented 50–100 times in a randomized order. In stage 3 (‘write’ stage), each holographic ensemble photoactivation was repeated 50 times in a randomized order. **b**, PSTHs of holographic evoked activity in directly photoactivated neurons. Four distinct neural ensembles were targeted—I_C1_-encoders, I_C2_-encoders, BR and TL segment responders and BL and TR segment responders. **c**, PSTHs of holographic evoked responses in nonphotoactivated neurons (>50 μm from all targets in each ensemble), for neurons that are visually responsive to the corresponding I_C_ image vs. nonresponsive neurons (24 sessions from 4 mice; mean ± s.e.m. across neurons). Baseline activity, defined as activity −1 to 0 s relative to holography onset, was subtracted for each neuron. **d**,**e**, Decoders trained on visual evoked activity (0–1 s relative to visual onset) were used to classify holography-evoked activity (0–1 s relative to holography onset). Only the holographic ensembles with ≥10 targets were analyzed. Nonphotoactivated neurons denote neurons >50 μm from all targets in each holographic ensemble. Box-and-whisker plots are formatted as follows: centerline, median; box limits, upper and lower quartiles; whiskers, 1.5× interquartile range; points, outliers. **d**, Holography trials are expected to be decoded as I_C1_ when targeting I_C1_-encoders, and I_C2_ when targeting I_C2_-encoders. Holography trials were decoded as expected when decoding all imaged neurons (left; *P* = 7.9 × 10^−7^ one-tailed Wilcoxon signed-rank test compared to chance across *n* = 32 IC-encoder ensembles). In addition, nonphotoactivated neurons were decoded as corresponding visual labels substantially above chance (right; *P* = 4.3 × 10^−5^; median performance = 0.3720, Q1–Q3 = 0.2870–0.5750). *P < 0.05. **e**, Holography trials are expected to be decoded as I_C1_ when targeting BR + TL segment responders, and I_C2_ when targeting BL + TR segment responders. Holography trials were decoded as expected when decoding all imaged neurons (left; *P* = 0.0029 one-tailed Wilcoxon signed-rank test compared to chance across *n* = 37 segment responder ensembles). However, nonphotoactivated neurons were not decoded as the corresponding visual label (right; *P* = 0.3254; median performance = 0.2280, Q1–Q3 = 0.1680–0.3535). Holo., holographic; stim., stimulation; vis., visual; resp., response.

One possibility is that V1 IC-encoders broadcast sensory inference signals through neural pattern completion within V1L2/3. This could promote sensory inference by selectively strengthening IC representations. Another possibility is that the segment responders of the inducing segments directly drive IC-encoders, resulting in neural pattern completion. To test these ideas, we selectively photoactivated ensembles of IC-encoders and segment responders using 2p holographic optogenetics (*n* = 24 sessions from four mice; same dataset as Fig. 4). We developed an ‘all-optical read-write’ experimental pipeline consisting of three stages (Fig. 5a). In stage 1 (the ‘read’ stage), we recorded the visual responses of several thousand V1L2/3 neurons to the IC image set. In stage 2 (the ‘online analysis’ stage), we analyzed the visual response properties of every neuron in the field-of-view to identify IC-encoders and segment responders. In stage 3 (the ‘write’ stage), we holographically stimulated distinct functional ensembles in the absence of visual inputs, while simultaneously imaging the same field-of-view. Holographically driven activity of four holographic ensembles is shown in Fig. 5b; I_C1_-encoders, I_C2_-encoders, BR and TL segment responders (that is, segments in I_C1_), BL and TR segment responders (that is, segments in I_C2_). With this approach, we could test whether selectively photoactivating ensembles of IC-encoders or segment responders was sufficient to drive IC representation on their own. If so, this would imply that during normal visual activity, pattern completion circuits in V1 facilitate IC inference.

**Extended Data Fig. 8:**
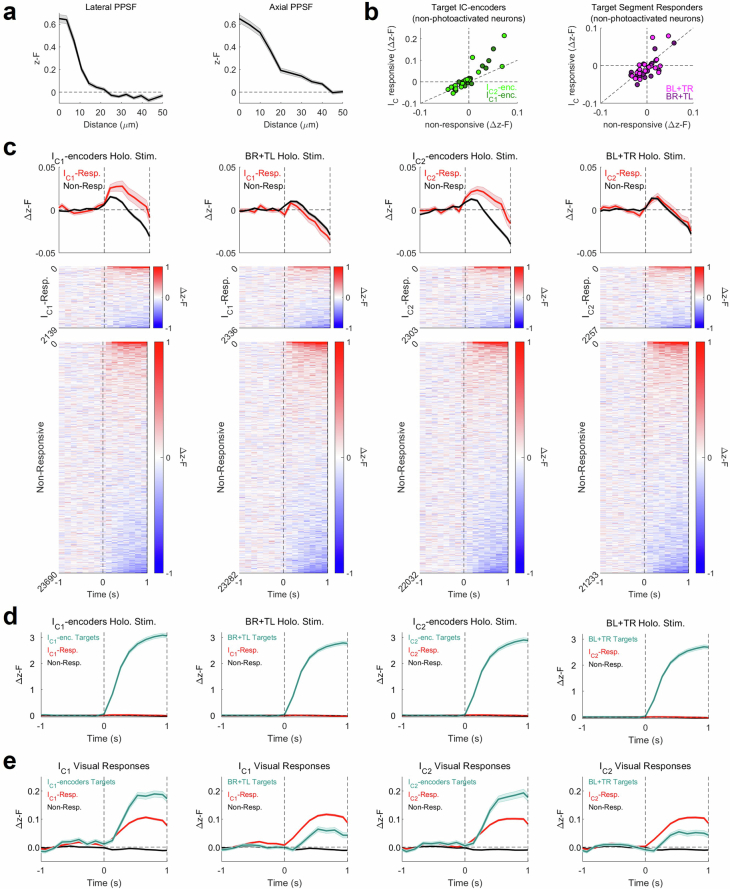
Holography evoked activity. **a**, Lateral (left) and axial (right) physiological point spread functions (PPSF) of the two-photon holographic photo-stimulation system, measured in GCaMP6m–ChRmine expressing neurons (mean ± s.e.m. across n = 294 neurons). **b**, Holography evoked responses in nonphotoactivated neurons (>50 μm from all targets in each ensemble), for neurons that are visually responsive to the corresponding I_C_ image (x axis) vs. nonresponsive neurons (y axis) (24 sessions from 4 mice). Holography-evoked response was measured during the photoactivation window (0–1 s relative to holography onset), and baseline activity was subtracted (−1 to 0 s relative to holography onset). Left, targeting IC-encoders evoked significantly larger activity in I_C_-responsive neurons than nonresponsive neurons (p = 0.0023 one-tailed Wilcoxon signed-rank test across n = 32 holographic ensembles). Right, targeting segment responders did not evoke a larger activity in I_C_-responsive neurons compared to nonresponsive neurons (p = 0.9660 across n = 37 holographic ensembles). **c**, PSTH of holography evoked responses. Baseline activity was subtracted as in **b**. Top, holography-driven PSTH averaged across all neurons in each group (mean ± s.e.m. across neurons; I_C_-responsive in red, nonresponsive in black). Bottom, holography-driven PSTH shown as a heatmap for each neuron in each group. **d**, Same as top panels in **c** (mean ± s.e.m. across neurons), but also showing targeted neurons in each holographic ensemble (cyan). Activity in directly photoactivated neurons is an order of magnitude higher than the activity in nonphotoactivated neurons. **e**, PSTH of visual evoked responses, for the same group of neurons shown in **d** (mean ± s.e.m. across neurons; directedly targeted neurons in cyan, nonphotoactivated I_C_-responsive neurons in red, nonphotoactivated non-I_C_-responsive neurons in black).

We sought to assess the output of photoactivated ensembles within V1L2/3 by examining the nonphotoactivated neurons in the field-of-view, excluding any cell in the vicinity of holographic targets that could have been directly photoactivated by the holographic light (Extended Data Fig. 8a). We compared the activity of I_C_-responsive neurons to non-I_C_-responsive neurons. I_C_-responsive neurons respond to I_C_ images, and they include both IC-encoders (which respond to I_C_ but not L_C_ images) and segment responders (which respond to both I_C_ and L_C_ images). If any of the holographic ensembles strengthened IC representations through neural pattern completion, we would expect them to synaptically drive neurons that are visually responsive to the I_C_ image differentially from those that are not I_C_ responsive. Consistent with prior reports, photoactivating a sparse ensemble of excitatory neurons in V1L2/3 led to a prolonged suppression of the nonphotoactivated population on average (Fig. 5c and Extended Data Fig. 8b,c)^26^. When targeting IC-encoders, this suppression was preceded by a selective activation of I_C_-responsive neurons during the photoactivation window (0–1 s relative to holography onset). Inspecting individual neurons revealed that the biphasic response can be decomposed into a relatively fast activation of a subset of neurons, and a slower suppression of a different subset of neurons (Extended Data Fig. 8c). This indicates substantial heterogeneity among the holographic evoked activity of nonphotoactivated neurons. Despite this heterogeneity, targeting IC-encoders evoked substantially larger activity in I_C_-responsive neurons compared to non-I_C_-responsive neurons during the photoactivation window (*P* = 0.0023 one-tailed Wilcoxon signed-rank test across *n* = 32 holographic ensembles). On the contrary, targeting segment responders did not evoke differential activity in I_C_-responsive vs. non-I_C_-responsive neurons (*P* = 0.9660 across *n* = 37 holographic ensembles). Taken together, photoactivation of IC-encoders, but not segment responders, drove mean activity differences between I_C_-responsive and non-I_C_-responsive neurons among nonphotoactivated neurons (Fig. 5c and Extended Data Fig. 8b,c). These results suggest that IC-encoders, but not segment responders, might drive neural pattern completion.

Next, we used decoding to assess neural pattern completion more holistically. Pattern completion refers to the generation of the entire pattern given a partial pattern. Thus, we first sought to establish that the photoactivation constituted a distinctive partial pattern of the visual evoked activity. To this end, we trained the neural decoder to classify visually evoked neural activity patterns with all neurons in the field-of-view (I_C1_ vs. L_C1_ vs. L_C2_ vs. I_C2_, as in Fig. 3a). Then, we asked how this decoder, trained only on visual evoked activity, would classify the holographically evoked neural activity in the absence of any visual inputs. As expected, the decoder classified the coactivation of I_C1_-encoders as the I_C1_ image and the coactivation of I_C2_-encoders as the I_C2_ image. In addition, it classified the coactivation BR + TL segment responders (that is, the inducing segments in I_C1_) as the I_C1_ stimulus, and the coactivation of BL + TR segment responders (that is, the inducing segments in I_C2_) as the I_C2_ stimulus. These results demonstrate that the holographic optogenetic activation of these ensembles successfully emulated partial patterns of visual stimulus-specific neural activity (Fig. 5d,e, left; mean ± s.e.m. = 18 ± 2 IC-encoders and 22 ± 3 segment responders were targeted; analysis was restricted to holographic ensembles with *n* ≥ 10 targets).

**Extended Data Fig. 9:**
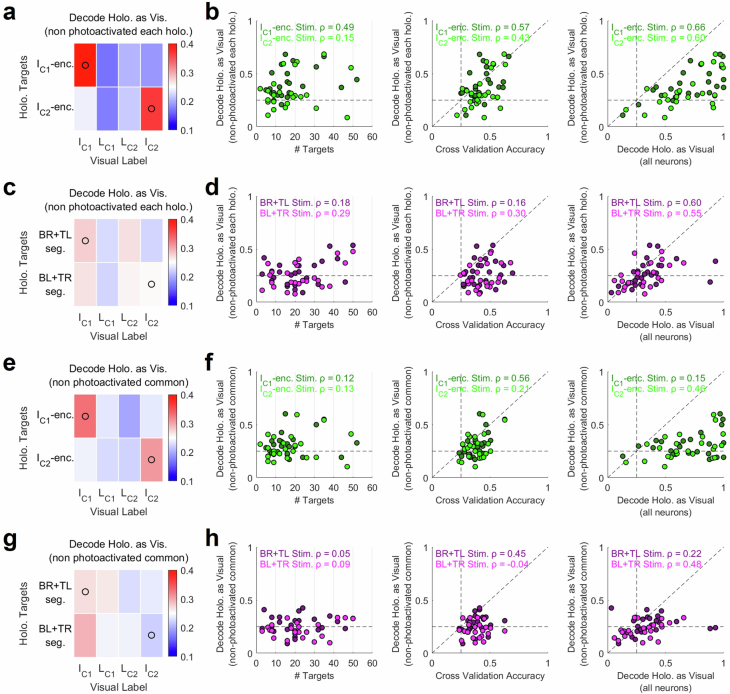
Holography evoked activity is decoded with decoders trained on visual evoked activity. In **a**–**d**, decoders were constructed with nonphotoactivated neurons, separately for each holographic ensemble (>50 μm from all targets in each ensemble; 24 sessions from 4 mice expressing GCaMP6m–ChRmine in V1 neurons). **a**, Holography-evoked response patterns are classified into the image that evoked the most similar visual response patterns. The expected classification given the holographic ensemble is marked with ‘o’. Only the holographic ensembles with ≥10 targets were analyzed (n = 17 I_C1_-encoder ensembles, n = 15 I_C2_-encoder ensembles). **b**, The proportion of holography trials decoded as the expected visual label (y axis), as a function of the number of targets in each holographic ensemble (left); tenfold cross-validation accuracy of the decoder trained on visual responses (middle); and the proportion of holography trials decoded as the expected visual label when decoder was trained on all neurons (right) (ρ denotes the Spearman correlation across holographic ensembles; n = 24 I_C1_-encoder ensembles, n = 24 I_C2_-encoder ensembles). **c**, Same as **a**, but for segment responder ensembles (n = 19 BR + TL segment responder ensembles, n = 18 BL + TR segment responder ensembles). **d**, Same as **b**, but for segment responder ensembles (n = 24 BR + TL segment responder ensembles, n = 24 BL + TR segment responder ensembles). **e**–**h**, Decoders were trained on neurons that were commonly nonphotoactivated across four holographic ensembles; I_C1_-encoders, I_C2_-encoders, BR + TL segment responders and BL + TR segment responders (>50 μm from all targets across these four ensembles). Otherwise, same as **a**–**d**.

Equipped with this approach, we asked if selective photoactivation of these ensembles could drive pattern completion within the local V1L2/3 network. If IC-encoders have a privileged role in amplifying sensory inferences within V1, they may drive decodable representations of I_C_ in the nonphotoactivated population. Indeed, the decoder constructed with nonphotoactivated neurons was able to classify IC-encoder photoactivation trials as the corresponding I_C_ image (Fig. 5d, right, and Extended Data Fig. 9a,b,e,f). In fact, decoding holographically driven activity with a decoder trained on visual trials showed a performance that approached the cross-validation accuracy of held-out visual trials for the same decoder (mean ± s.e.m. performance for decoding holography trials as visual image labels was 0.4029 ± 0.0296, whereas cross-validation accuracy was 0.4818 ± 0.0165; Extended Data Fig. 9b). This implies that holographic-driven activity was able to emulate visual-driven activity with remarkable accuracy.

In contrast, photoactivating a similar number of segment responders did not evoke decodable activity patterns (Fig. 5e, right, and Extended Data Fig. 9c,d,g,h). These effects were consistent regardless of whether the decoder was constructed from nonphotoactivated neurons for each holographic ensemble (Fig. 5d,e, right, and Extended Data Fig. 9a–d), or from a common pool of nonphotoactivated neurons (Extended Data Fig. 9e–h). Thus, photoactivating IC-encoders, but not segment responders, was sufficient to drive V1 activity patterns resembling those visually evoked by the corresponding I_C_ image even in the absence of any visual stimulus. The pattern completion effects of IC-encoders correlated with the number of targets in the holographic ensemble (Extended Data Fig. 9b). Of note, in these experiments, we photoactivated nearly every IC-encoder within the imaged population. Thus, the decoder classified the induced network activity pattern only from the non-IC-encoders.

**Extended Data Fig. 10:**
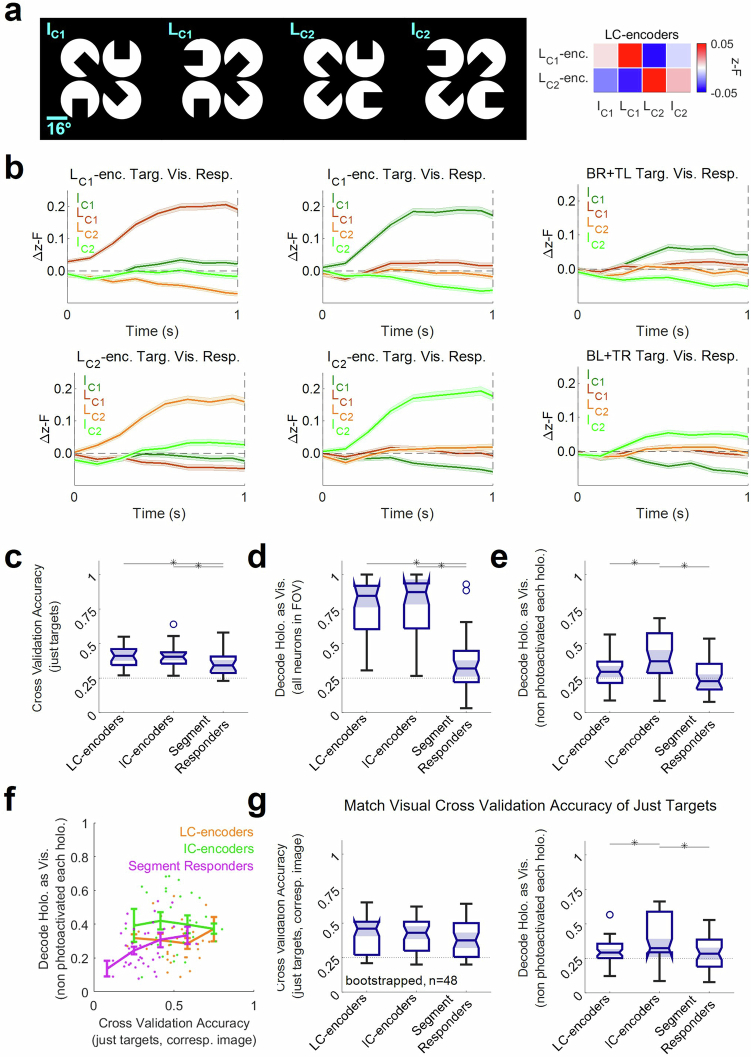
LC-encoders are not as effective as IC-encoders at driving recurrent pattern completion in V1 layer 2/3, despite containing equivalent amounts of visual information. **a**, LC-encoders are defined as neurons that respond to either L_C1_ or L_C2_, but not to either I_C1_ or I_C2_ (p < 0.05 Kruskal–Wallis test across responses to blank vs. I_C1_ vs. L_C1_ vs. L_C2_ vs. I_C2_, post hoc Tukey–Kramer test). In **b**–**g**, LC-encoders were targeted in two distinct holographic ensembles; L_C1_-encoder photoactivation is expected to be decoded as L_C1_, and L_C2_-encoder photoactivation is expected to be decoded as L_C2_ (n = 24 sessions from 4 mice expressing GCaMP6m–ChRmine in V1 neurons). **b**, Visual responses of neurons in each holographic ensemble. Mean ± s.e.m. across n = 428 L_C1_-encoders, n = 292 L_C2_-encoders, n = 354 I_C1_-encoders, n = 372 I_C2_-encoders, n = 424 BR + TL segment responders, n = 409 BL + TR segment responders. In **c**–**e**, only the holographic ensembles with ≥10 targets were analyzed (n = 35, 32 and 37 ensembles for LC-encoders, IC-encoders and segment responders, respectively). Box-and-whisker plots are formatted as follows: centerline, median; box limits, upper and lower quartiles; whiskers, 1.5x interquartile range; circles, outliers. **c**, To compare visual information contained in holographic ensembles, we compared the tenfold cross-validation accuracy of visual responses for targeted neurons on corresponding visual trials; LC-encoder ensembles on L_C_ trials (L_C1_/L_C2_-encoders on L_C1_/L_C2_ trials, respectively), IC-encoder ensembles on I_C_ trials (I_C1_/I_C2_-encoders on I_C1_/I_C2_ trials, respectively) and segment responder ensembles on I_C_ trials (BR + TL/BL + TR segment responders on I_C1_/I_C2_ trials, respectively; Kruskal–Wallis test p = 2.0 × 10^−8^, followed by Bonferroni–Holm corrected Wilcoxon rank-sum tests *p < 0.05). **d**, Decoders constructed with all neurons were trained on visual evoked activity patterns and used to classify holography evoked activity patterns. Only the holographic ensembles with ≥10 targets were analyzed (n = 35, 32 and 37 ensembles for LC-encoders, IC-encoders and segment responders, respectively; Kruskal-Wallis test p = 2.4 × 10^−11^, followed by Bonferroni–Holm corrected Wilcoxon rank-sum tests *p < 0.05). **e**, Decoders were constructed with nonphotoactivated neurons for each holographic ensemble (Kruskal–Wallis test p = 0.0012, followed by Bonferroni–Holm corrected Wilcoxon rank-sum tests *p < 0.05). **f**, Relationship between visual information contained in each holographic ensemble (x axis; shown in **c**), and the decoding of holographically driven activity in nonphotoactivated neurons (y axis; shown in **e**). Cross-validation accuracy of holographic ensembles (x axis) was divided into 6 equidistant bins between 0 and 1, and mean ± s.e.m. decoder performance (y axis) of each bin was plotted for three groups of holographic ensembles (orange: n = 35 LC-encoder ensembles, green: n = 32 IC-encoder ensembles, magenta: n = 37 segment responder ensembles). **g**, For the three groups of holographic ensembles, cross-validation accuracies of targets were matched by matching the number of samples in each x-axis bin shown in **f**. Specifically, in each of the three bins where all three groups had data, we chose the group with the highest number of samples, and bootstrapped data in the other two groups to match the sample number. As a result, the bootstrapped distributions were matched in cross-validation accuracies of targets (left; n = 48 ensembles in each group, Kruskal–Wallis test p = 0.5811). When comparing the decoding performance of holographically driven activity in nonphotoactivated neurons among these bootstrapped distributions, we again see the highest performance when targeting IC-encoders (right; n = 48 ensembles in each group, Kruskal–Wallis test p = 0.0022, followed by Bonferroni–Holm corrected Wilcoxon rank-sum tests *p < 0.05). Box-and-whisker plots are formatted as in **c**–**e**.

As a further test of the distinct capacity of IC-encoders to drive pattern completion, we photoactivated ‘LC-encoders’, which we defined as neurons that selectively respond to one of the L_C_ images but not to any of the I_C_ images (Extended Data Fig. 10a,b). LC-encoders were substantially less potent at driving pattern completion of L_C_ representations compared to IC-encoders, despite being matched in targeted numbers, visual selectivity and contribution to decoding performance (Extended Data Fig. 10c–e). Moreover, the optogenetically driven recurrent activity of nonphotoactivated neurons was substantially more likely to be decoded as the corresponding I_C_/L_C_ image when photoactivating IC-encoders compared to LC-encoders and segment responders, even when the amount of visual information contained in the holographic ensembles were matched (Extended Data Fig. 10f,g). Together, these results indicate that IC-encoders are distinctively potent at driving pattern completion of IC representations within V1L2/3.

### IC encoding is mediated by a looped circuit in the visual cortical hierarchy

**Fig. 6:**
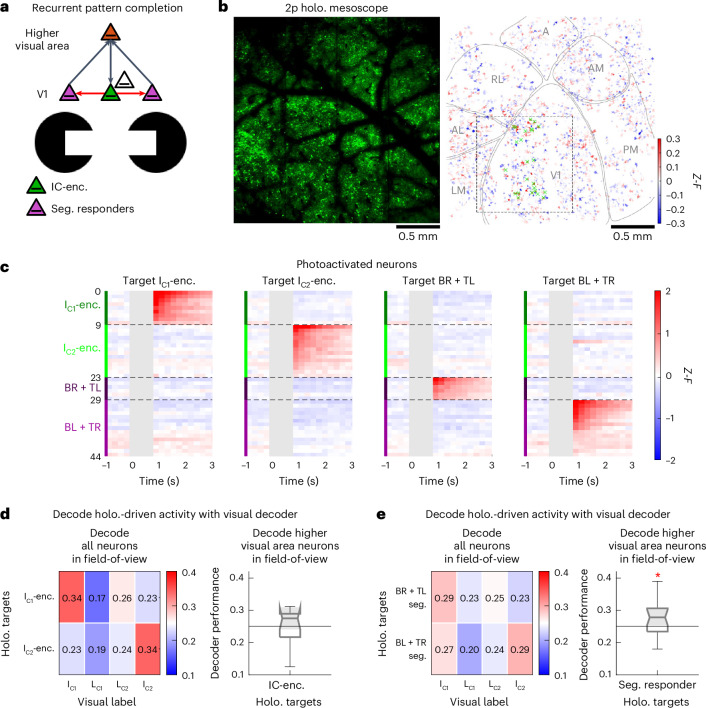
Mesoscale 2p holographic optogenetic interrogation reveals the corticocortical loop that encodes ICs. **a**, IC circuit schematic. V1 segment responders relay feedforward information to higher visual areas; V1 IC-encoders receive feedback from higher visual areas and locally broadcast the top-down information to the rest of the V1L2/3 network. **b**, Left, mesoscope field-of-view (2.4 × 2.4 mm^2^; Vglut1-CreXAi203 mouse; 11 sessions across two mice). Right, ‘influence map’ of holography-evoked responses in an example 2p holographic mesoscope experiment (targets are marked with green ‘x’). Holographic field-of-view (dotted rectangle) was positioned over V1. Putative visual area borders are demarcated. **c**, As in Fig. 5, four holographic ensembles were targeted in V1L2/3; I_C1_-encoders, I_C2_-encoders, BR and TL segment responders and BL and TR segment responders. **d**,**e**, Decoders trained on visual evoked activity (0–1 s relative to visual onset) were used to classify holography-evoked activity (0–1 s relative to holography offset; 11 sessions across two mice expressing GCaMP7s–ChroME in excitatory neurons). For each holographically targeted ensemble, only the sessions with ≥10 targets were analyzed. Nonphotoactivated neurons denote neurons >25 μm from all targets in each holographic ensemble. Box-and-whisker plots are formatted as follows: centerline, median; box limits, upper and lower quartiles; whiskers, 1.5× interquartile range; points, outliers. **d**, V1 IC-encoder ensembles were targeted for photoactivation. Left, when decoding all neurons in the field-of-view, holography-evoked activity patterns were decoded as the corresponding visual images (*n* = 11 IC-encoder ensembles; mean ± s.e.m. = 16 ± 1 targets, *P* = 0.0010 one-tailed Wilcoxon signed-rank test compared to chance). When decoding nonphotoactivated neurons in higher visual areas (all neurons in the field-of-view outside of V1), holography-evoked activity patterns were not decoded as corresponding visual images (*P* = 0.3501; median performance = 0.2750, Q1–Q3 = 0.2169–0.2893). **e**, Same as in **d**, when targeting V1 segment responder ensembles for photoactivation (*n* = 22 segment responder ensembles, mean ± s.e.m. = 21 ± 1 targets; left decode all neurons, *P* = 0.0047, right decode nonphotoactivated neurons in higher visual areas, *P* = 0.0457; median performance = 0.2780, Q1–Q3 = 0.2340–0.3060). *P < 0.05.

The 2p holographic optogenetics experiments described above suggest a key new component to the conventional model of IC encoding (Fig. 6a). Consistent with the conventional model, our conceptual model proposes that segment responders receive bottom-up sensory signals and IC-encoders receive top-down inference signals^16,17^. Our data suggest a critical addition to this conceptual model—IC-encoders locally broadcast the top-down inference signal within the V1L2/3 network. Taken together, this leads to the prediction that segment responders have a primary role in feedforward propagation, while IC-encoders have a primary role in local reinforcement of inferential signals (Fig. 6a). If this is true, photoactivating segment responders should promote IC representations in higher visual areas, while IC-encoders should not. To test this hypothesis, we needed the technical ability to read out not just the local effects of holographic stimulation, but also the downstream effects. To this end, we used a new platform that we recently developed, a 2p holographic mesoscope^18^. This platform allowed us to holographically stimulate V1 neurons while observing network effects in several downstream higher visual areas simultaneously (Fig. 6b,c).

First, as above, decoding from all neurons in the mesoscopic field-of-view confirmed that photoactivation of V1 IC-encoder and segment responder ensembles evoked activity patterns that partially emulated those evoked by the corresponding images (analysis was restricted to holographic ensembles with *n* ≥ 10 targets; Fig. 6d, left; *n* = 11 IC-encoder ensembles with mean ± s.e.m. = 16 ± 1 targets; Fig. 6e, left, *n* = 22 segment responder ensembles with 21 ± 1 targets). Next, we decoded only from nonphotoactivated neurons in higher visual areas to test whether photoactivating IC-encoders or segment responders recreated IC representations in downstream regions. In stark contrast to pattern completion effects within V1L2/3 (Fig. 5d,e, right), V1 segment responders, but not V1 IC-encoders, drove IC representation in higher visual areas (Fig. 6d,e, right, and Supplementary Fig. 6). These data demonstrate that V1 segment responders drive feedforward communication of the bottom-up sensory information to higher visual areas, while V1 IC-encoders drive pattern completion within V1L2/3 that can reinforce this process. Taken together, our data establish recurrent looped connectivity among V1 IC-encoders, V1 segment responders and higher visual areas.

## Discussion

We combined large-scale electrophysiology, 2p imaging, 2p holographic optogenetics and 2p holographic mesoscope to establish a direct, causal link between neural pattern completion in the primary V1 and sensory inference. We identified a subpopulation of visual cortical neurons that specifically encode ICs as emergent features of the image. Next, by leveraging the large numbers of simultaneously recorded neurons, we developed a multivariate approach to identify patterns of neural activity that encode sensory inferences. Using this new ‘inference decoding’ paradigm, we found strong representations of IC inference in L2/3 of V1 and LM. In contrast, thalamo-recipient L4 in V1 did not represent IC inference. Therefore, V1L2/3 is the lowest area in the visual hierarchy to be involved in IC inference computation. Further analysis of inference decoding in V1L2/3 revealed that IC-encoders mediate the representation of the inferred illusory contour. Strikingly, we found that 2p holographic stimulation of IC-encoders drove neural pattern completion within V1L2/3, in the absence of visual inputs. While segment responders did not induce IC representation within V1L2/3, further examination of the visual cortical hierarchy using the 2p holographic mesoscope revealed that segment responders drove IC representations in higher visual areas.

Prior literature emphasized the role of top-down feedback in generating IC inference signals in V1 (refs. ^7,14,15^). Although this top-down feedback explains why IC responses emerge in V1, it does not elucidate their potential role, if any. Our findings fill in this gap by highlighting pattern completion within V1 as a critical missing step in existing models of IC encoding^16,17^. This pattern completion in V1 may help to ensure that only visual signals that match prior expectations are selectively reinforced during recurrent activity between V1 and higher visual areas.

Our experimental demonstration of recurrent looped connectivity in the visual hierarchy confirms a critical prediction from computational studies of IC inference. Previous studies have found that conventional convolutional neural networks (CNNs) with purely feedforward architectures fail to ‘perceive’ ICs, but adding in recurrent looped connectivity leads to the emergence of IC inference^27–29^. Thus, our results provide definitive biological evidence for a key prediction from artificial neural network (ANN) studies—recurrent looped connectivity in the visual hierarchy is essential for IC inference.

Several studies have shown that cortical stimulation of a small number of neurons could trigger highly structured patterns of neural activity, or even goal-directed behavior, in the absence of any sensory inputs^30–33^. Such structured patterns can even emerge during spontaneous activity^34,35^. These studies revealed that cortical circuits are primed for pattern completion, likely due to structured connectivity. A prominent example of such structured connectivity is the like-to-like connectivity, that is, enhanced connectivity between neurons encoding coherent sensory information^36–40^. However, no prior work has studied neocortical pattern completion, nor its underlying connectivity, in the context of sensory inference. Our study suggests a vital function for neocortical pattern completion in sensory inference.

We propose that the recurrent pattern completion underlying IC encoding is ubiquitous in active perceptual processing of the external world. Sensory systems are constantly faced with incomplete or ambiguous sensory information. In these situations, successful perception depends on sensory inference. For example, humans are capable of fast and accurate object recognition even when objects are occluded^41^. Similar to ICs, top-down predictions about occluded objects are thought to be implemented by feedback connections from higher sensory areas^21,23,42^. Reinforcement of top-down predictions in lower sensory areas through recurrent pattern completion likely also has a critical role in visual object recognition during occlusion^41^. More generally, feedback-driven pattern completion in lower cortical areas may be a universal mechanism of sensory inference. This pattern completion may generate recurrent activity spanning lower and higher sensory cortices, iteratively refining inference computation by selectively reinforcing neural activity patterns that match prior expectations. Taken together, holistic percepts can emerge despite sensory ambiguity through recurrent pattern completion in the neocortical sensory hierarchy.

## Methods

### Animals

All experiments were performed in C57/B6 mice of both sexes, aged 6 weeks and older. Imaging experiments in Figs. 1–3 were conducted with CaMKII-tTA;tetO-GcaMP6s mice (V1L2/3 and mesoscope L2/3 data), and Scnn1a-Tg3-Cre;Ai162(TIT2L-GC6s-ICL-tTA2)-D mice (V1L4 data). Neuropixels experiments were conducted with Sst-IRES-Cre;Ai32(RCL-ChR2(H134R)_EYFP) mice (*n* = 10) and PV-IRES-Cre;Ai32(RCL-ChR2(H134R)_EYFP) mice (*n* = 4). The 2p holographic optogenetics experiments in Figs. 4 and 5 were conducted with AAV8-hSyn-GCaMP6m-p2A-ChRmine-Kv2.1-WPRE injected in PV-IRES-Cre;RCL-tdTomato mice (*n* = 2) and AAV9-2YF-hSyn-DIO-GcaMP6m-P2A-ChRmine-Kv2.1-WPRE in Emx1-Cre mice (*n* = 2). Furthermore, 2p holographic mesoscope experiments in Fig. 6 were conducted with Vglut1-Cre;Ai203 mice (Ai203 refers to a transgenic line expressing the transgene TITL-st-ChroME-GCaMP7s-ICL-nls-mRuby3-IRES2-tTA2; *n* = 2). All experiments on animals were conducted with approval of the Animal Care and Use Committee of the University of California, Berkeley (imaging and 2p holographic optogenetics experiments) and the Allen Institute’s Institutional Animal Care and Use Committee (Neuropixels experiments).

### Surgery

Mice were anesthetized with isoflurane (2%) and mounted on a stereotaxic apparatus. Buprenorphine (0.05 mg kg^−1^, analgesic) and dexamethasone (2 mg kg^−1^, anti-inflammatory) were injected subcutaneously. Body temperature was maintained at 36.5 °C. After hair removal and disinfection with 70% ethanol and 5% iodine, the scalp was removed and the fascia was retracted. A circular craniotomy of 3–5 mm was made on the left hemisphere using a biopsy punch (Robbins Instruments) and/or a dental drill (Foredom) with a 0.5 mm drill bit (FST Item 19007-05). Glass coverslips of 3–5 mm diameter were placed on top of the craniotomy and sealed with dental cement (C&B Metabond). These glass coverslips, or ‘windows’, were made beforehand by gluing two layers of round cover glass (for example, 1 × 3 mm + 1 × 5 mm, Warner Instruments, 1 thickness) with Norland Optical Adhesive 71 (UV-cured). Finally, a titanium headplate was fixed to the skull with dental cement (C&B Metabond). The dental cement was coated in a layer of black oxide to mitigate light leakage during 2p imaging.

For 2p holographic optogenetics experiments, we injected the GCaMP6m-p2A-ChRmine-Kv2.1 virus during the window implant surgery (500 nl per two depths and two sites). Injection speed was 100–250 nl min^−1^, and after injection in each site, pipette was held in place for 5 min. Injection sites were centered at 2.7 mm lateral, 0.2 mm anterior relative to lambda and injection depths were 120 µm and 250 µm.

### Neuropixels experiments

The Neuropixels data were acquired at the Allen Institute as part of the OpenScope project that allows the community to apply for observation on the Allen Brain Observatory platform (https://alleninstitute.org/division/mindscope/openscope/). The Neuropixels pipeline at the Allen Institute is described in detail in ref. ^43^ and Durand et al.^44^, as well as the online white paper^45^. Briefly, the mouse underwent headplate implantation and craniotomy surgery, in a procedure similar to the one described above. The 5-mm craniotomy was sealed with a glass coverslip, the bottom of which was coated with silicone to reduce adhesion to the brain surface. The glass coverslip was held in place with Vetbond and Kwik-Cast (World Precision Instruments) until the day of the recording (several weeks later). Intrinsic signal imaging was performed to identify visual area boundaries^46^. Based on the identified visual areas, an insertion window was designed for each mouse. Mice were habituated to head fixation and visual stimulation over a period of 2 weeks.

On the day of recording, the cranial coverslip was removed and replaced with the insertion window containing holes aligned to six cortical visual areas. Mice were allowed to recover for 1–2 h after insertion window placement, before being head-fixed in the recording rig.

Six Neuropixels probes were targeted to each of the six visual cortical areas (V1, LM, RL, AL, PM and AM). Probes were doused with CM-DiI (1 mM in ethanol; Thermo Fisher Scientific, V22888) for post hoc ex vivo probe localization. Each probe was mounted on a 3-axis micromanipulator (New Scale Technologies). The tip of each probe was aligned to its associated opening in the insertion window guided by an Indian Statistical Institute map of the visual system. The operator then moved each probe into place with a joystick, with the probes fully retracted along the insertion axis, approximately 2.5 mm above the brain surface. The probes were then manually lowered one by one to the brain surface until spikes were visible on the electrodes closest to the tip. After the probes penetrated the brain to a depth of around 100 μm, they were inserted automatically at a rate of 200 μm min^−1^ (total of 3.5 mm or less in the brain). After the probes reached their targets, they were allowed to settle for 5–10 min.

Neuropixels data were acquired at 30 kHz (spike band, 500-Hz high-pass filter) and 2.5 kHz (LFP band, 1,000-Hz low-pass filter) using the Open Ephys GUI^43^. Videos of the eye and body were acquired at 30 Hz. The angular velocity of the running wheel was recorded at the time of each stimulus frame, at approximately 60 Hz.

The spike-band data are median-subtracted, first within-channel to center the signal around zero, then across channels to remove common-mode noise. The median-subtracted data file is sent to the Kilosort2 MATLAB package (https://github.com/mouseland/Kilosort2)^47^, which applies a 150-Hz high-pass filter, followed by whitening in blocks of 32 channels. Kilosort2 models this filtered, whitened data as a sum of spike ‘templates’. The shape and locations of each template are iteratively refined until the data can be accurately reconstructed from a set of N templates at M spike times. Finally, Kilosort2 output is curated to remove putative double-counted spikes and units with artefactual waveforms. All units not classified as noise were packaged into Neurodata Without Borders files and analyzed in this paper.

As our focus was on excitatory neurons, we analyzed regular-spiking (RS) units in the Neuropixels dataset, defined as spike-sorted single units with extracellular spike trough-to-peak width of ≥0.4 ms. For brevity, we referred to RS units as ‘neurons’.

### The 2p imaging experiments

The 2p imaging experiments presented in Figs. 1–3 were conducted in two setups. V1L2/3 and V1L4 imaging was done on a 2p microscope (Neurolabware with Scanbox GUI) with a Ti:sapphire laser (Chameleon Ultra II, Coherent), an electrotunable lens for multiplane imaging (Optotune), 8 kHz galvo-resonant scanner and a Nixon ×16 magnification 12.5-mm focal length 0.8 NA water immersion objective. Typical image settings were as follows: field-of-view size 1,223 × 675 μm^2^ (796 × 512 pixels), four planes spaced 50 μm apart sampled at volumetric frame rate 7.715 Hz, imaging wavelength 930 nm.

Mesoscope 2p imaging was done on 2p random access mesoscope (Thorlabs with ScanImage GUI), equipped with a Ti:sapphire laser (Mai Tai, Spectra Physics), remote-focusing unit consisting of an objective and a mirror mounted on a voice coil, 12 kHz galvo-resonant scanner and Jenoptiks/Thorlabs water immersion objective with 21 mm focal length and 0.6 excitation NA and 1.0 collection NA. Typical image settings were as follows: field-of-view size 3,031 × 2,965 μm^2^ (3,040 × 1,484 pixels), frame rate 3.139 Hz, imaging wavelength 920 nm.

During post hoc analysis, motion correction and source extraction were done using Suite2p^48^. Suite2p output putative regions of interest (ROIs) that correspond to neuronal somas, and these ROIs were manually inspected to keep only those that looked like cell bodies. Neuropil signal was subtracted from each ROI with a coefficient of 0.7 (*F*_*C*_ = *F*_ROI_ − 0.7 × *F*_neu_). *Z*-*F* was calculated over each continuous block of recording as the *z*-scored *F*_*C*_, that is, ((*F*_*C*_ − mean(*F*_*C*_))/std(*F*_*C*_)). Δ*Z*-*F* was calculated by subtracting baseline period activity (gray-screen period). For brevity, we referred to manually curated ROIs in the 2p data as ‘neurons’.

### The 2p holographic optogenetics experiments

For 2p holographic optogenetics experiments presented in Fig. 5, three-dimensional (3D) scanless holographic optogenetics with temporal focusing (3D-SHOT) path was implemented in a Sutter MOM (Movable Objective Microscope, Sutter Instrument Co., with ScanImage GUI), as described previously^26,49–52^. Ti:sapphire laser (Chameleon Ultra II, Coherent) was used for 2p imaging, and femtosecond fiber laser (Satsuma HP2, 1030 nm, 2 MHz, 350 fs; Amplitude Systems) was used for 2p holographic stimulation. The holography path included a blazed diffraction grating for temporal focusing (600 l mm^−1^, 1,000 nm blaze; Edmund Optics, 49-570 or 33010FL01-520R; Newport), and a rotating diffuser to randomize the phase pattern and expand the beam. Both the imaging path and the holography path had spatial light modulators (SLM; HSP1920, 1,920 × 1,152 pixels; Meadowlark Optics). The imaging path SLM enabled optical axial focusing. The SLM was conjugated to the X resonant galvo and optical axial focusing was achieved by loading Fresnel-lens-like phase patterns on the SLM for each desired axial depth. The high refresh rate provided by the overdriven SLM allowed the acquisition speed to be limited only by the number of planes (no settling time or frame drop). The holography path SLM was used to display the holographic phase mask, calculated using the Gerchberg–Saxton algorithm to place several cell-sized diffraction-limited spots in 3D target positions^50^. A zero-order block was placed in the holography path. The imaging path and the holography path were merged by a polarizing beam splitter before the microscope tube lens and the objective (Olympus ×20 magnification, 9-mm focal length, 1.0 NA water immersion objective). To limit imaging artifacts introduced by the femtosecond laser, the femtosecond laser output was synchronized to the scan phase of the galvo-resonant scanner (8 kHz) using an Arduino Mega, gated to be only on the edges of every line scan.

Precise alignment of the imaging path and the holography path is achieved by a calibration procedure described previously^26,49,50^. Briefly, we place a thinly coated fluorescent slide on a substage camera to image both the imaging planes and the holograms at parametrically varied SLM coordinates. Then, the 3D coordinate transformation from imaging coordinates on each plane to SLM coordinates is computed through the ‘polyfitn’ function in Matlab.

Before each experiment, the alignment is confirmed for each remote-focused plane by ‘burning holes’ on a fluorescent slide (photobleaching). The intended imaging coordinates are displayed on ScanImage (Vidrio) as Integration ROIs. The match between the intended coordinates and the burnt hole positions confirms the validity of the current calibration.

As described in the ‘Results’, the ‘all-optical read-write’ 2p holographic optogenetic experiments were conducted in three stages. First was the ‘read’ stage, where we image visual responses of neurons in the V1L2/3 field-of-view. Second was the online analysis stage, where we ran Suite2p on the imaging data collected in the first stage and identified functional groups of neurons (IC-encoders, segment responders). Third was the ‘write’ stage, where we holographically stimulated these functionally identified neuron groups. Throughout all stages of the experiment, the mouse was head-fixed under the objective, and the imaging conditions were kept constant (same field-of-view of size 980 × 980 μm^2^, same set of four planes spaced 30 μm apart, with each plane being imaged every four frames at 7.525 Hz volumetric frame rate, imaging wavelength of 920 nm).

For fast online analysis, we converted ScanImage Tiff files into four h5 files, generating one h5 file per plane. Then, we ran Suite2p for each h5 file independently, in parallel (‘multiprocessing’ package in Python). On an eight-core CPU (Intel Core i7 10700K 3.8 GHz) with 128 GB RAM, online Suite2p of a 25 min imaging data (512 × 512 pixels) took eight minutes. Then, all ROIs identified by the Suite2p were analyzed in Matlab to identify functional groups of neurons (IC-encoders and segment responders). Finally, Suite2p output ‘stat.med’ was used as the coordinate of the ROIs of interest. These Suite2p coordinates were converted to SLM coordinates per the calibration described above.

Because IC-encoders are sparse, we targeted every neuron identified as IC-encoders during the online analysis step. For segment responders, we balanced the number of segment responders in the four groups (BR, BL, TL and TR). For example, if the BR-segment responders had the lowest number (N), we chose the top N most responsive segment responders from the other three groups. Responsiveness was quantified as the area under receiver operating curve in the ideal observer analysis discriminating between neural responses on inward vs. outward segment image trials. Mean number of targets in each holographic ensemble were as follows: 17 I_C1_-encoders, 18 I_C2_-encoders, 22 BR + TL segment responders 22 BL + TR segment responders.

After determining the holography groups with online analysis, holograms containing all targets in each group were calculated using the Gerchberg–Saxton algorithm^50^. The holograms were diffraction efficiency corrected to achieve homogeneous power delivery of 7 mW to all targeted cells. Holographic stimulation on each trial consisted of ten pulses at 10 Hz with 10 ms pulse width, and the ITI was 4.5 s. Stimulation of each holography group was repeated 50 times.

During post hoc offline analysis, Suite2p was run over the entire recording, including stage 1 (read stage, visual block) and stage 3 (write stage, holography block). On average, 1,968 ROIs were classified as putative neurons in the four-plane volumetric field-of-view. Targets were identified as the neuron closest to each target coordinate. Because online analysis does not entail manual curation of the ROIs, a target may not correspond to a neuron. Thus, a target was considered valid only if the closest neuron was within 10 µm, which left 81.5% of targets (mean number of valid targets for each holography group was 15 I_C1_-encoders, 16 I_C2_-encoders, 18 BR + TL segment responders and 17 BL + TR segment responders). Of these, holographic stimulation evoked substantial activation in 91.6% of valid targets (mean number of stimulated targets for each holography group was 14 I_C1_-encoders, 14 I_C2_-encoders, 16 BR + TL segment responders and 16 BL + TR segment responders). Nonphotoactivated neurons were defined as neurons >50μm from all holographic targets in each ensemble; distance threshold informed by the axial physiological point spread function in Extended Data Fig. 8a.

### The 2p holographic mesoscope experiments

We developed the 2p holographic mesoscope by incorporating the 3D-SHOT into the Thorlabs 2p random access mesoscope. Full details are reported in a separate paper^18^. The holographic field-of-view was placed over V1 (gray-dotted rectangle in Fig. 6b, right). The imaging field-of-view encompassed V1, LM, RL, AL, PM and AM (2,400 × 2,400 m^2^). The imaging and holographic field-of-view was fixed throughout each recording session. The experimental pipeline was identical to the 2p holographic optogenetics experiments (Fig. 5a). During holographic ensemble stimulation, each target received 50 mW, and each stimulation consisted of ten pulses of 10 ms duration at 16 Hz. Zero-power ‘blank’ trials were interleaved. Each trial type was repeated 100 times with an ITI of 5 s.

### Visual stimulation monitor placement and retinotopy

All Neuropixels recordings were made according to the standardized Neuropixels visual coding pipeline. Visual stimuli were generated using custom scripts based on PsychoPy62 and were displayed using an ASUS PA248Q LCD monitor, with 1,920 × 1,200 pixels (55.7-cm wide, 60-Hz refresh rate). Stimuli were presented monocularly, and the monitor was positioned 15 cm from the right eye of the mouse and spanned 120° × 95° of visual space. Monitor placement was standardized across rigs such that the mouse’s right eye gaze would typically be directed at the center of the monitor.

For all 2p experiments, visual stimuli were generated using custom Matlab scripts based on Psychtoolbox-3 (ref. ^53^). In the standard 2p imaging setup, a 19-inch Samsung monitor was placed 15 cm from the mouse’s right eye (1,280 × 1,024 pixels, 37.6 × 30.1 cm width and height, 60-Hz refresh rate). In the mesoscope 2p imaging setup and the 2p all-optical read-write setup, Adafruit Qualia 9.7″ DisplayPort Monitor was placed 8 cm from the right eye of the mouse (2,048 × 1,536 pixels, 19.7 × 14.8 cm width and height). The backlight of the Adafruit monitor was triggered by the galvo-resonant scanner such that the monitor would emit light only during the mirror turnaround time, similar to the gating of the holographic stimulation described above (12 kHz in the mesoscope 2p imaging setup, 8 kHz in the 2p all-optical read-write setup). In addition, in all 2p experiments, electrical tape was applied between the objective and the mouse’s headplate to prevent monitor light from contaminating 2p imaging.

In all 2p experiments, retinotopic mapping was used to place the monitor such that the most common receptive field of the field-of-view would align with the center of the monitor. In the mesoscope 2p imaging, retinotopic mapping was additionally used to determine visual area boundaries using visual field sign maps^54,55^. After settling on the imaging field-of-view at the beginning of each 2p experiment, retinotopic mapping was conducted with 16° rectangular drifting grating patches appearing in one of 25 positions tiling an 80° × 80° visual degree grid. These patches had spatial frequency of 0.04 cycles per degree and a temporal frequency of 2 Hz, cycling through eight directions (0°/45°/90°/135°/180°/225°/270°/315°) over the course of 2 s, followed by a 1 s gray-screen intertrial interval (ITI). Each patch position was presented with three to five repeats, with randomized trial order.

### Visual stimulus

After retinotopic mapping, visual stimulation consisted of three blocks—IC block, receptive field mapping block and size tuning block. Trial order was randomized within each block.

In the IC block, images described as I_C_, L_C_, I_RE_, T_RE_ were shown (IC configuration 1; Extended Data Fig. 1b). In Neuropixels experiments and a subset of 2p imaging experiments, these images were also shown rotated 315° clockwise in a separate block (IC configuration 0; Extended Data Fig. 1b). The diameter of the white circles was 30 visual degrees, and the distance between the centers of the diagonally placed white circles were 46 visual degrees (that is, the gap between diagonal circles were 16 visual degrees). The black bar length on each inducer segment was 16 visual degrees, such that the IC support ratio in I_C_ images would be 2/3. Edges delimiting the real bar in the real edge images (I_RE_/T_RE_/X_RE_) were at least 2° thick, which is well over the grating acuity threshold (0.55 cycles per degree) and, in fact, close to the maximum sensitivity of mouse vision (0.2 cycles per degree)^19^. Throughout each IC block, the four white circles stayed in place even during the ITI. In Neuropixels recordings, each image was presented for 0.4 s followed by 0.4 s ITI. In IC configuration 1, each image was repeated at least 50 times, and I_C_ and L_C_ images were repeated 400 times; in IC configuration 0, each image was repeated 30 times. Generally, in 2p experiments, each image was presented for 1 s followed by 0 s ITI. Each image was repeated at least 50 times, and I_C_ and L_C_ images were repeated 400–500 times. In 2p holographic optogenetics experiments, each image was presented for 1 s followed by 0 s ITI. Each image was repeated 50–100 times. In 2p recordings in Fig. 1, each image was presented for 1 s followed by a variable ITI of 1–2 s. Each image was repeated 10 times in 9 sessions and 50 times in 20 sessions.

In the receptive field mapping block, circular patches of drifting gratings with a fixed diameter of 16 visual degrees were presented in nine different locations, as shown in Extended Data Fig. 3a; one patch was positioned at the center of the monitor, the rest were positioned at 16 visual degrees distance from the center in 6/4.5/3/1.5/12/10.5/9/7.5 o’clock positions. Drifting gratings had a spatial frequency of 0.04 cycles per degree and a temporal frequency of 2 Hz. The drifting gratings had 100% contrast and were presented on a gray background. On each trial, a circular patch of drifting grating appeared in one of nine positions and cycled through several directions spaced 45° apart, drifting in each direction for 0.25 s (4/8 directions lasting 1/2 s in Neuropixels/2p experiments, respectively). Presentation at each location was repeated ten times, with 0 s ITI.

In the size tuning block, circular patches of drifting gratings were presented with a fixed position at the center of the monitor, in different sizes of 0/4/8/16/32/64 visual degrees. Spatial frequency, temporal frequency and contrast of drifting gratings were identical to the receptive field mapping block. On each trial, a circular patch of drifting grating at a given size appeared in one of eight directions (0°/45°/90°/135°/180°/225°/270°/315°). In Neuropixels experiments, the presentation lasted 0.25 s followed by 0.5 s ITI, and each combination of size and direction was repeated 10 times. In 2p imaging experiments, the presentation lasted 1 s followed by 0 s ITI, and each combination of size and direction was repeated 10 times. The size tuning block was skipped in 2p holographic optogenetics experiments.

### Eye tracking analysis

For results presented in Extended Data Fig. 2, we limited the analysis to trials where the mouse was fixating (fixed-gaze trials). Video of the mouse eye during the neural recording session was analyzed using DeepLabCut^56^. Resting pupil position was determined as the mode of the pupil position. Fixed-gaze trials were defined as trials where pupil position was within eight visual degrees of the resting pupil position throughout the duration of the trial.

### Definition of functional subsets of neurons

Exclusively center-responsive neurons were defined as neurons that exclusively responded to circular grating patches in the center position, corresponding to the illusory gap region, and not to any of the other adjacent positions (Fig. 1; Wilcoxon rank-sum test between trials with circular grating patches in each position and gray-screen trials, Bonferroni–Holm correction for multiple comparisons). In the Neuropixels data, there were *n* = 18 exclusively center-responsive neurons of 1,804 V1 RS units. In the 2p imaging data, there were *n* = 310 exclusively center-responsive neurons of 18,576 V1L2/3 neurons. The low proportion of neurons is expected from the strict criteria we imposed.

IC-encoders were defined as neurons that respond to one of the I_C_ images, and to neither of the L_C_ stimuli (Kruskal–Wallis test across responses to ‘blank’ (four white circles) vs. I_C1_ vs. L_C1_ vs. L_C2_ vs. I_C2_ with Tukey–Kramer correction for multiple comparisons). Across ICwcfg0 and ICwcfg1, 159 of 1,804 V1 RS units were identified as IC-encoders in the Neuropixels dataset; 1,569 of 18,576 V1L2/3 excitatory neurons were identified as IC-encoders in the 2p dataset.

Segment responders were defined as neurons that selectively responded to inward-pointing inducer segments in each inducer position. For example, BR-segment responders are defined as neurons that respond substantially more to In_BR_ than Out_BR_ (Wilcoxon rank-sum test; Fig. 2a,b).

### Decoding analysis

For all decoding analyses presented in the main figures (Figs. 3–6), we used a support vector machine (SVM) with a linear kernel. First, we constructed the trial-by-trial response matrix (trials x neurons) as the response averaged over the visual stimulus presentation period (firing rate for Neuropixels data, *z*-*F* for 2p data). We divided the trials with the training trial types (I_C1_/L_C1_/L_C2_/I_C2_) into a training set (90%) and a testing set (10%). For tenfold cross-validation, there were ten splits of training/testing sets, and the ten sets of test trials were nonoverlapping. On each of the ten iterations, the trial-by-trial response matrix was *z*-scored based on the training set, that is, response matrix was subtracted by the mean of training trials and divided by the s.d. of training trials. On each iteration, SVM was trained using the ‘fitcecoc’ function in MATLAB. Cross-validation accuracy was computed as the SVM’s prediction accuracy on the testing set, averaged across ten iterations. The inference decoding performance and the holography-evoked activity decoding performance were also measured as the average prediction performance of the ten SVMs.

The holography-evoked response matrix, used as the input to the decoder in Fig. 5d,e, was calculated as the normalized *z*-*F* in the 1 s period following the onset of the holographic stimulation. Normalization entailed subtracting the mean and dividing by the s.d. across all holography trials. To analyze the effects of photoactivation in the rest of the network, we analyzed only the nonphotoactivated neurons that were at least 50 μm away from all holographic targets in each ensemble. As shown by the physiological point spread function (PPSF; Extended Data Fig. 8a), all direct influence of holographic light dissipates by 50 μm.

The holographic mesoscope data were analyzed similarly, but with two adjustments. First, we took the 1 s period following the offset of the holographic stimulation. As described above, the holographic stimulation was confined to the turnaround period of the resonant mirror. The 2p random access mesoscope patches together multiple imaging regions, and holographic artifacts appear on either side of each imaging region. The region affected by the holographic artifact is correspondingly larger. Thus, we did not analyze the holographic stimulation period. Second, nonphotoactivated neurons were defined as neurons >25 μm from targets in each holographic ensemble; distance threshold was informed by the lateral physiological point spread function in this case because the mesoscopic field-of-view was imaged at a single depth.

In Figs. 5 and 6, nonphotoactivated neurons are defined for each holographic ensemble. Hence, the decoders for nonphotoactivated neurons consist of different sets of neurons for each ensemble. To assess whether these differences in decoder population affected the results, we also tried decoding off of a common pool of nonphotoactivated neurons (Extended Data Fig. 9e–h). The results remain the same as when decoding different sets of nonphotoactivated neurons for each holographic ensemble (Extended Data Fig. 9a–d). As such, the differential effects of distinct holographic ensembles are not an artifact of decoding from slightly different subsets of neurons.

In Supplementary Fig. 3, we compared the decoding performance of several different kinds of decoders. SVMs were trained with different kernels—linear vs. quadratic polynomial vs. radial basis function. In addition, a fully connected artificial neural network (ANN) was trained using Pytorch, similar to the decoding described in ref. ^57^. The ANN had an input layer with number of nodes matching the number of neurons, two hidden layers each with 100 nodes, and one output layer with number of nodes matching the number of training trial types (that is, 4 nodes, each corresponding to I_C1_/L_C1_/L_C2_/I_C2_). Softmax function was applied to the output layer. The ANN was trained for 50,000 epochs with the Adam optimizer and the CrossEntropyLoss loss function. All decoders underwent tenfold cross-validation, as described in detail above.

### Cross-correlogram analysis

The cross-correlogram is defined as:

$${\rm{Cross}}{\hbox{-}}{\rm{correlogram}}\left(\tau \right)=\,\frac{\mathop{\sum }\nolimits_{t=1+{\tau }_{\max }}^{L-{\tau }_{\max }}{x}_{1}(t){x}_{2}(t+\tau )}{\sqrt{{\lambda }_{1}{\lambda }_{2}}}$$

where *x*_1_ and *x*_2_ are spike trains of two units across the IC configuration 1 block (72 min, 1-ms bins), *L* is the length of the spike trains, *λ*_1_ and *λ*_2_ are the net spike count across each spike train and *τ* is the time lag between the spike trains.

To account for cofluctuations in rate, smoothed cross-correlogram was calculated from spike trains smoothed with a 25-ms window. Then, we calculated the corrected cross-correlogram by subtracting the smoothed cross-correlogram from the original cross-correlogram.

To determine whether there was a putative excitatory connection between a pair of units, peaks in corrected cross-correlogram were found in 1–10-ms window. The connection was deemed substantial if the peak value exceeded the threshold, where the threshold was calculated as sevenfold of the cross-correlogram flank s.d. (between ±50 and 100 ms from zero).

### Statistics and reproducibility

Nonparametric two-sided tests are used unless otherwise noted. Adjustments for multiple comparisons have been noted wherever they were applied. When applicable, we used cross-validation to avoid overfitting.

No statistical method was used to predetermine sample size, but we used an equivalent or larger sample size than similar studies^18,26,58,59^. Trial order within each block was randomized. All data exclusions are described along with exclusion criteria and the rationale for exclusion. Analysis of Neuropixels data was performed blind to collection and spike sorting. Manual curation of 2p imaging data (Suite2p) was performed blind to visual and holography-evoked responses.

### Reporting summary

Further information on research design is available in the Nature Portfolio Reporting Summary linked to this article.

## Online content

Any methods, additional references, Nature Portfolio reporting summaries, source data, extended data, supplementary information, acknowledgements, peer review information; details of author contributions and competing interests; and statements of data and code availability are available at 10.1038/s41593-025-02055-5.

## Supplementary information

Supplementary Information Supplementary Results, Supplementary Discussion and Supplementary Figs. 1–8.

Reporting Summary

## Data Availability

Neuropixels dataset is available at: https://dandiarchive.org/dandiset/000248/ (see also https://openscopedatafront.web.app/table). Additional postprocessed data are available at 10.24433/CO.6659254.v2.

## Code availability

Analysis codes for generating all figures are available at 10.24433/CO.6659254.v2.
