# Supplementary material for: Recurrent pattern completion drives the neocortical representation of sensory inference
Source: Nat Neurosci. 2025 Sep 15;28(11):2319–29. doi: 10.1038/s41593-025-02055-5 (PMC12586158; doi:10.1038/s41593-025-02055-5)
Supplement: Supplementary file 1 — Supplementary Results, Supplementary Discussion and Supplementary Figs. 1–8. [file 41593_2025_2055_MOESM1_ESM.pdf]

nature neuroscience

Supplementary information https://doi.org/10.1038/s41593-025-02055-5

Recurrent pattern completion drives the neocortical representation of sensory inference

In the format provided by the authors and unedited

Supplementary Results

IC-encoders can have receptive fields outside of the illusory gap region (Extended Data Fig. 3a-g). Combined with the finding that IC-encoders mediate the representation of IC inference, we would not expect inference signals in V1 layer 2/3 to be confined to neurons with receptive fields on the illusory gap region. To test this, we zeroed out all center-responsive neurons, defined as neurons that respond to visual contrast in the central 16 visual degree region (mean ± SEM across sessions 124±14 center-responsive neurons). Zeroing out all center-responsive neurons did not abolish the IC inference performance (Supplementary Fig. 5b). Thus, even neurons with receptive fields entirely outside of the illusory gap region can carry IC inference signals in V1 layer 2/3.

Our data establish looped connectivity between V1 IC-encoders, V1 segment responders, and higher visual areas. We asked how this previously unknown recurrent looped connectivity shapes visual responses to illusory contours (Supplementary Fig. 8a). Without this recurrent connectivity, segment responders are expected to respond equally to real and illusory bars, since the inducing segments are the same and hence the bottom-up inputs are the same. On the other hand, IC-encoders are expected to respond faster to real bars, since IC-encoders receive bottom-up inputs with real bars but not with illusory bars[1]. We hypothesized that due to the lateral connectivity between V1 IC-encoders and V1 segment responders, V1 segment responders would also respond faster to real bars than illusory bars (Supplementary Fig. 7-8). Indeed, both V1 IC-encoders and segment responders responded faster to real bars (T_{RE} images; V1 IC-encoders median 81ms, Q1 – Q3 70 – 94ms; V1 segment responders median 83ms, Q1 – Q3 72 – 94ms) than illusory bars (I_C images; V1 IC-encoders median 107ms, Q1 – Q3 72 – 240ms; V1 segment responders median 88ms, Q1 – Q3 76 – 106ms; Supplementary Fig. 7e, g). Importantly, that segment responders have different response latencies for illusory vs real bars is inconsistent with the notion that V1 has a passive role in IC inference (Supplementary Fig. 7a *left*); instead, the results are consistent with our proposed looped circuitry of IC-encoding (Supplementary Fig. 7a *right*).

Supplementary Discussion

Our study unveils a previously unknown step in IC encoding: IC-encoders drive pattern completion within V1 layer 2/3. Importantly, the pattern completion circuit within V1 layer 2/3 completes the loop between V1 and higher visual areas (Fig. 6a). More generally, a subset of neurons in lower cortical areas may mediate sensory inference by locally broadcasting top-down predictions via pattern completion. Further, sensory inference may be iteratively refined via the recurrent loop between V1 and higher visual areas, such that neural activity patterns that match prior expectations about the sensory world are selectively reinforced.

Prior work on illusory contours have mostly relied on receptive field-based analyses[1,2]. These papers have reported that neurons with receptive fields within the illusory gap region can show visual responses to illusory contours, a finding that we have replicated in Fig. 1 and Extended Data Fig. 2-3. In this paper, we focused primarily on IC-encoders, defined as neurons that respond selectively to images containing illusory bars (I_C images), but not to reshuffled versions of these images (L_C images). In other words, IC-encoders showed contextual responses that were selective to the global arrangement of image segments that induces the illusory contour. Interestingly, IC-encoders could have receptive fields outside of the illusory gap region (Extended Data Fig. 4). This is in line with mounting evidence suggesting that neurons in mouse V1 respond to contextual information outside of their receptive fields [3-5].

There is a possibility that L_C images might drive the illusion of an 'L' shape. Nevertheless, the possibility of the 'L' illusion does not affect any of our conclusions. First, the definition of IC-encoders is unaffected because they are defined to be specific to each unique illusory bar, rather than being defined as a general indicator of illusions. Second, T_{RE} inference decoding is unaffected because in the T_{RE} image, we outlined the bar that is illusory in the I_C image. Thus, the T_{RE} inference decoding assesses whether the illusory bar is represented in the I_C-evoked activity, independently of whether the 'L' contour is represented in the L_C-

evoked activity. Moreover, X_{RE} inference decoding, which does not use L_C stimuli at all, shows highly consistent results with T_{RE} inference decoding (Extended Data Fig. 7). In sum, our conclusions are unaffected by the possibility of the 'L' illusion in L_C images.

While looped connectivity between V1 and higher visual areas have been anatomically demonstrated[6,7], whether and how looped connectivity across the visual cortical hierarchy contributes to IC encoding has remained unknown. By comparison, our data is parsimonious with a tri-component loop consisting of feedforward, feedback and local connections within V1 layer 2/3 (Fig. 6a). As in prior models of IC-encoding[8,9], the neurons sending bottom-up feedforward projections from V1 to higher visual areas (segment responders) are distinct from the neurons receiving top-down feedback projections (IC-encoders) in our conceptual model (Supplementary Fig. 8a). The cross-correlogram analysis supports the notion that IC-encoders preferentially receive top-down inputs (Fig. 2d), although this analysis is correlative and may therefore be prone to biases[10]. The 2p holographic mesoscope experiments support the notion that V1 segment responders send feedforward projections to higher visual areas (Fig. 6e). Most importantly, the 2p holographic optogenetics data supports the centerpiece of our conceptual model (Fig. 5d); that local recurrent connectivity within V1 layer 2/3 closes the loop between V1 and higher visual areas.

We caution against a direct comparison between the photoactivation effects of IC-encoders versus segment responders. Segment responders were defined based on their responses to the inducing segments of the illusory bar, which are present in the I_C images. Even though this definition makes it likely that segment responders respond to I_C images, responsiveness to I_C images was not a prerequisite for segment responders. On the contrary, IC-encoders are, by definition, responsive to I_C images. As such, I_C image presentation entails a coactivation of IC-encoders, but coactivation of the inducing segment responders is not guaranteed. Despite these caveats, we can conclude from the 2p holographic optogenetics experiments that IC-encoders have a prominent role in driving local pattern completion (Fig. 5, Extended Data Fig. 10). Further, we can conclude from the 2p holographic mesoscope experiments that segment responders have a role in driving feedforward cortico-cortical sensory relay (Fig. 6).

Given the looped connectivity between V1 IC-encoders, V1 segment responders, and higher visual areas (Fig. 6a), one might ask why the holographic manipulation did not engage the entire loop. We speculate that this has to do with the number of photoactivated neurons. We estimate that we are imaging and manipulating roughly 2% of the local network (assuming around 100,000 neurons in mouse V1 layer 2/3 [11]). It is remarkable that manipulating such a small fraction of the relevant subpopulation can drive decodable patterns of neural activity. Justifiably, however, this holographic manipulation does not appear to be strong enough to elicit indirect downstream effects. Future advances that increase the scale of 2p holographic optogenetics could overcome this barrier.

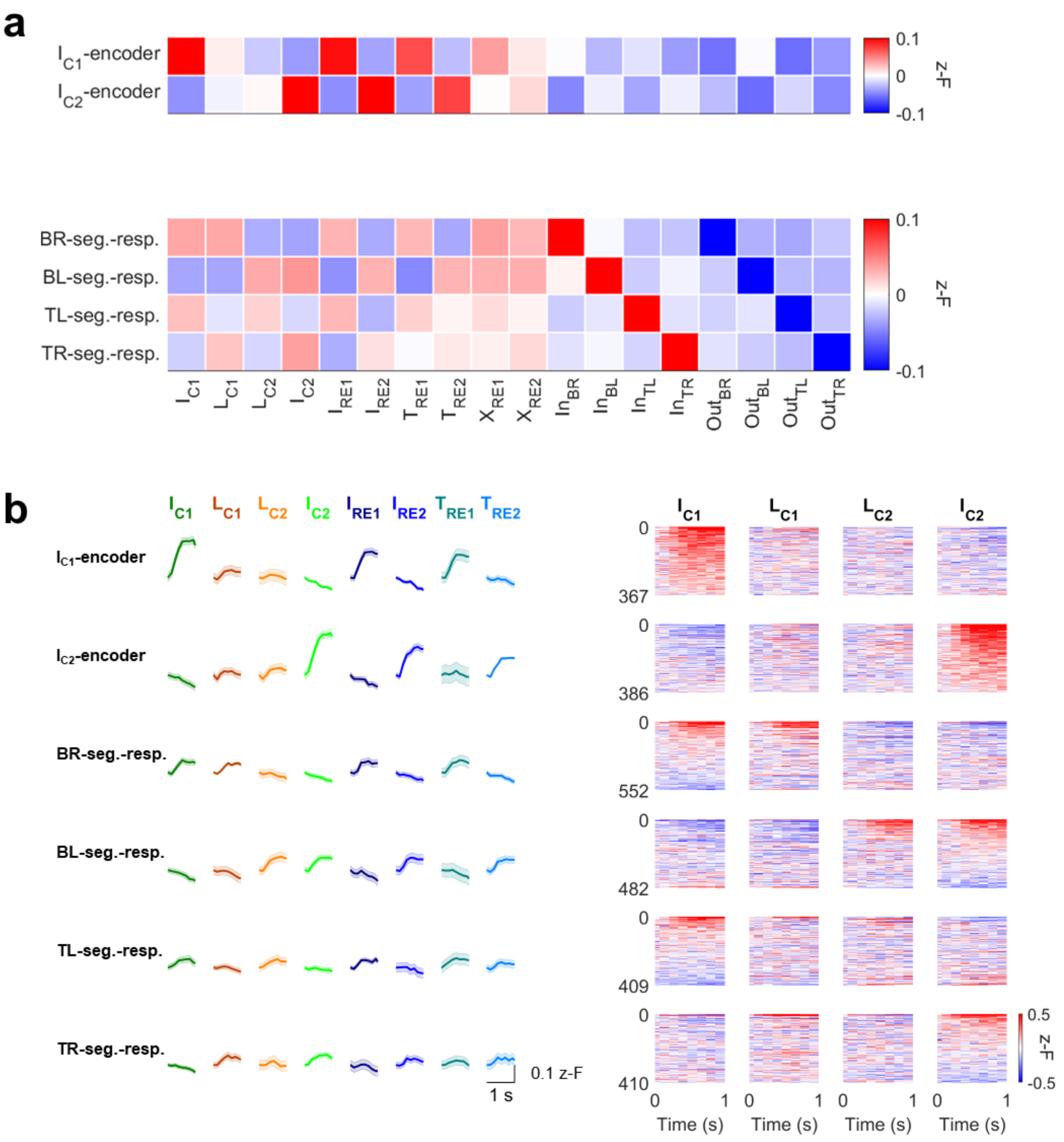

Supplementary Figure 1. Visual response properties of functionally defined groups of neurons.

a, Visual responses of I_{C1}-encoders (n=367), I_{C2}-encoders (n=386), bottom-right (BR) segment responders (n=552), bottom-left (BL) segment responders (n=482), top-left (TL) segment responders (n=409), top-right (TR) segment responders (n=410) (2p imaging of V1 layer 2/3 FOV; 24 sessions from 4 mice). I_{C1}/I_{C2}-encoders are neurons that respond to the I_{C1}/I_{C2} image but not to L_{C1} nor L_{C2} (i.e., defined as neurons with $p<0.05$ Kruskal-Wallis test across responses to blank vs I_{C1} vs L_{C1} vs L_{C2} vs I_{C2}, post-hoc Tukey-Kramer test). Segment responders respond more strongly to the inward segments in each position compared to the outward segments (i.e., defined as neurons with $p<0.05$ Wilcoxon rank-sum test).

b, Same data as **a**, shown as PSTHs. Evoked response traces are shown for the visual presentation window (0 –1s relative to sensory onset; *Left* shows across neuron mean ± SEM; *Right* shows heatmap with each neuron in each row, ordered by evoked activity on the relevant I_C image).

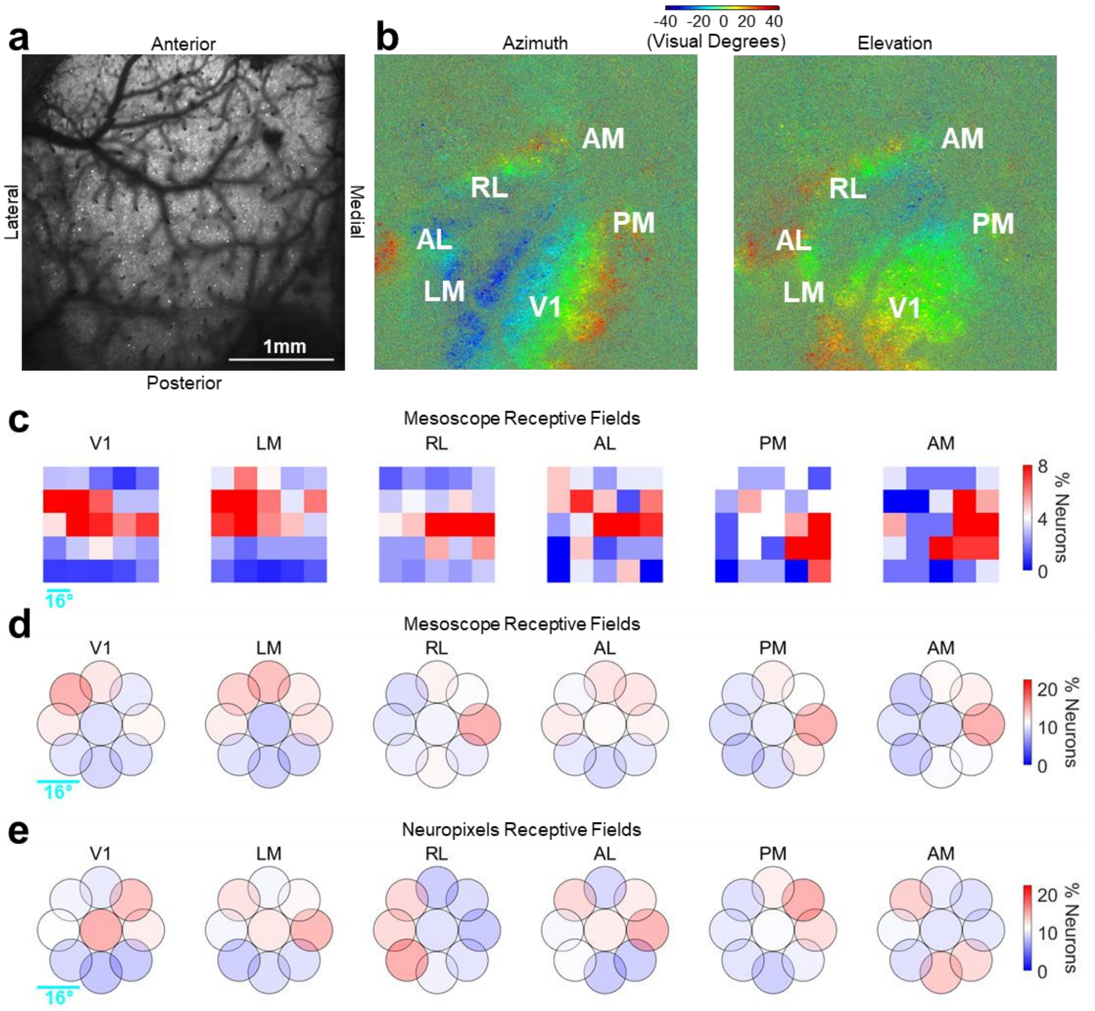

Supplementary Figure 2. Receptive field distributions of each visual area in the 2p mesoscope and Neuropixels datasets.

a, Mesoscope FOV (3031X2965 μm^2, 3040X1484 pixels; CaMKII-tTA;tetO-GcaMP6s mouse; n=19 sessions from 5 mice).

b, Receptive fields were mapped with a 5X5 grid of 16 visual degree square patches of drifting gratings. Each pixel in the FOV is color coded by the azimuth (*left*) or the elevation (*right*) of its receptive field position (i.e., the square patch position that evokes the largest response).

c, Receptive field distributions in the mesoscope dataset, when mapped with 5X5 grid of square patches. Only the neurons with significant receptive fields are represented ($p<0.05$ Kruskal-Wallis test across responses to different positions). Number of significant neurons out of all neurons recorded in each area is: n=1208/8893 for V1, n=361/2788 for LM, n=284/2682 for RL, n=82/631 for AL, n=75/1698 for PM, and n=56/678 for AM.

d, Receptive field distributions in the mesoscope dataset, when mapped with 16 visual degree circular patches in 9 different positions. Number of significant neurons out of all neurons recorded in each area is: n=1289/8893 for V1, n=389/2788 for LM, n= 270/2682 for RL, n=101/631 for AL, n=105/1698 for PM, and n=67/678 for AM.

e, Receptive field distributions in the Neuropixels dataset, when mapped with 16 visual degree circular patches in 9 different positions. Number of significant RS units out of all RS units recorded in each area is n=904/1804 for V1, n=562/1091 for LM, n=433/1561 for RL, n=550/1241 for AL, n=341/1731 for PM, and n=269/1369 for AM.

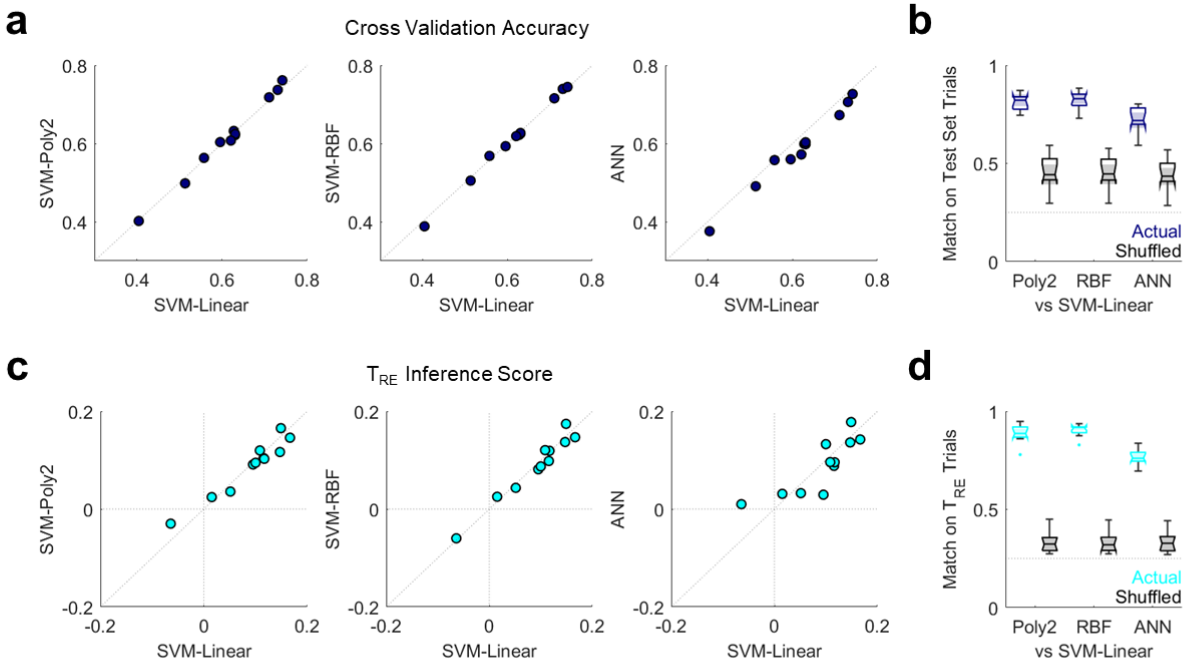

Supplementary Figure 3. Decoder prediction is consistent across decoder types.

a, Cross validation accuracy comparison across n=11 sessions, between SVM with a linear kernel (SVM-Linear, x-axis) vs SVM with a quadratic polynomial kernel (SVM-Poly2, y-axis on left panel), or radial basis function kernel (SVM-RBF, y-axis on center panel), or fully connected artificial neural network (ANN, y-axis on right panel).

b&d, Box-and-whisker plots are formatted as follows: center line, median; box limits, upper and lower quartiles; whiskers, 1.5x interquartile range; circles, outliers.

b, Proportion of trials in the held-out test set that had matching decoder predictions, between SVM-Linear decoder and the other three decoder types (n=11 sessions). For comparison, match proportion was also calculated with the trial order shuffled within each trial type (I_{C1}, L_{C1}, L_{C2} and I_{C2}); for each session, match proportions were averaged across 1000 shuffles.

c, T_{RE} inference score comparison across n=11 sessions, where T_{RE} inference score was defined as $P(T_{RE}\rightarrow I_C) - P(T_{RE}\rightarrow L_C)$.

d, Proportion of T_{RE} trials that have matching decoder predictions, compared to match proportion when trials are shuffled within each trial type (T_{RE1} and T_{RE2}; n=11 sessions).

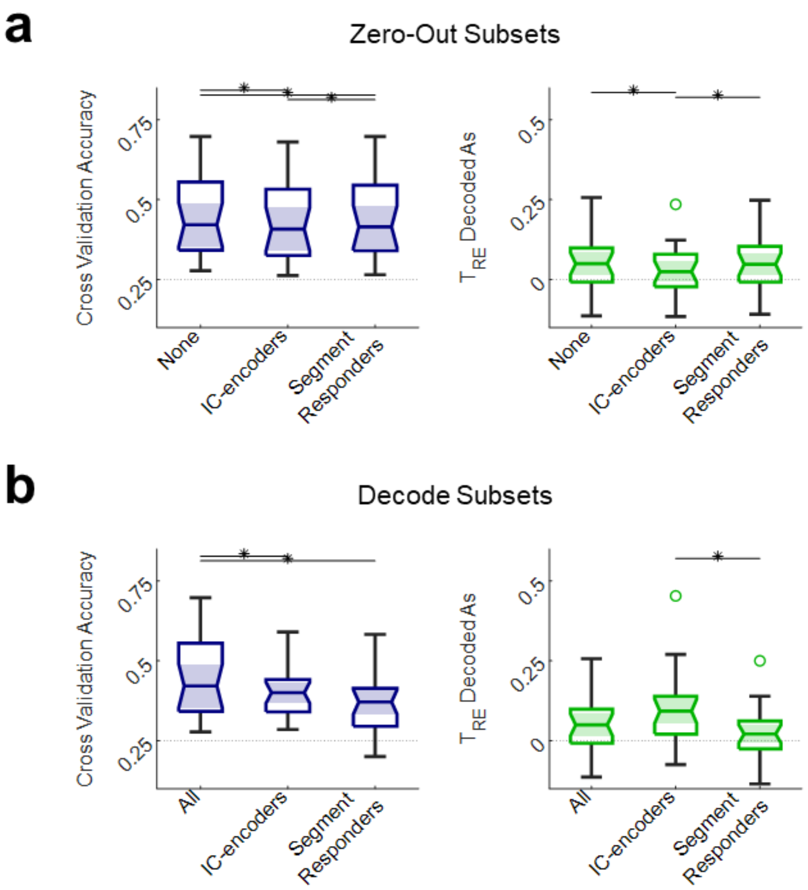

Supplementary Figure 4. Statistical comparison of neuronal subsets for Fig. 4 results.

a, Decoder performance when zeroing out the indicated subsets of neurons in the input to the decoder (n=24 sessions from 4 mice; *left*: cross-validation accuracy Friedman test across subsets $p=1.1\times10^{-5}$, followed by Bonferroni-Holm corrected Wilcoxon signed rank tests between pairs of subsets $*p<0.05$; *right*: T_{RE} inference score Friedman test across subsets p=0.0019, followed by Bonferroni-Holm corrected Wilcoxon signed rank tests between pairs of subsets $*p<0.05$).

b, Decoder performance when decoding only from the indicated subsets of neurons (same subsets as **b**; Friedman test p=0.0025 for cross-validation accuracy and p=0.0208 for T_{RE} inference score; n=24 sessions from 4 mice, $*p<0.05$ Bonferroni-Holm corrected Wilcoxon signed rank tests between pairs of subsets).

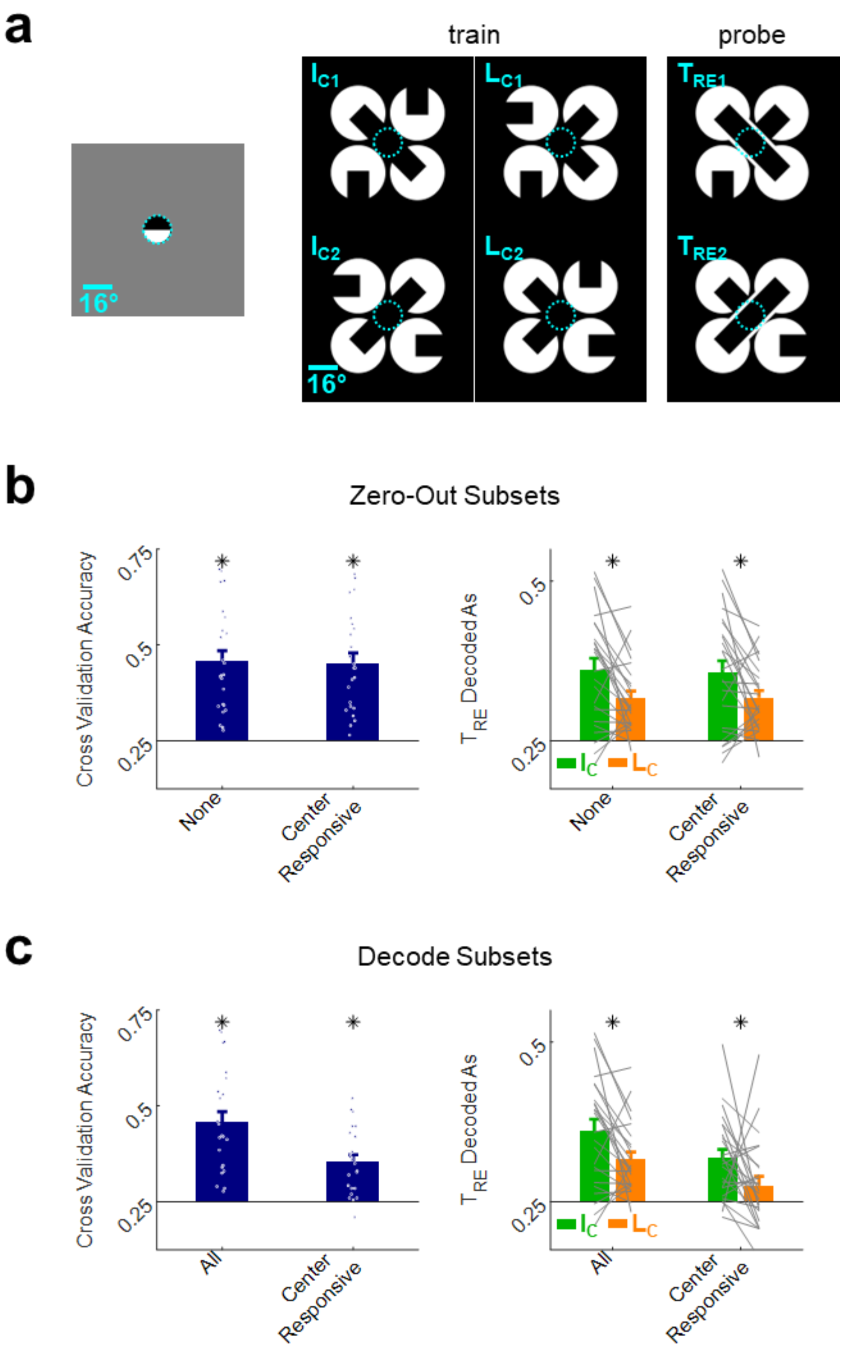

Supplementary Figure 5. Illusory contour inference signal is not confined within the illusory gap region.

a, Center responsive neurons were defined as neurons that responded to a 16 visual degree circular patch of drifting gratings in the center position ($p<0.05$ Wilcoxon rank-sum test compared to activity on gray-screen trials). Drifting gratings in each position were presented in multiple orientations in a fixed sequence (0°, 45°, 90°, 135°), such that neurons with any orientation preference would be driven.

b-c, Same dataset as Fig. 4 (mean ± SEM across n=24 sessions from 4 mice). *Left*: *$p<0.05$ Wilcoxon signed-rank test compared to cross-validation accuracy chance performance of 0.25. *Right*: *$p<0.05$ Wilcoxon signed-rank test comparing fraction of I_C vs L_C trials decoded as the corresponding T_{RE}.

b, Zeroing out center responsive neurons did not abolish the T_{RE} inference score, suggesting that inference signal is not confined to center responsive neurons (cross validation accuracy Wilcoxon signed-rank test $p=1.8\times10^{-5}$, 1.8×10^{-5}; T_{RE} inference score Wilcoxon signed-rank test $p=0.0177$, 0.0425 for 'none' and 'center responsive', respectively).

c, Decoding just the center responsive neurons also results in a significant T_{RE} inference score. Together, these results suggest that center responsive neurons contain some, but not all, of the illusory contour inference signal (cross validation accuracy Wilcoxon signed-rank test $p=1.8\times10^{-5}$, 4.4×10^{-5}; T_{RE} inference score Wilcoxon signed-rank test $p=0.0177$, 0.0119 for 'all' and 'center responsive', respectively).

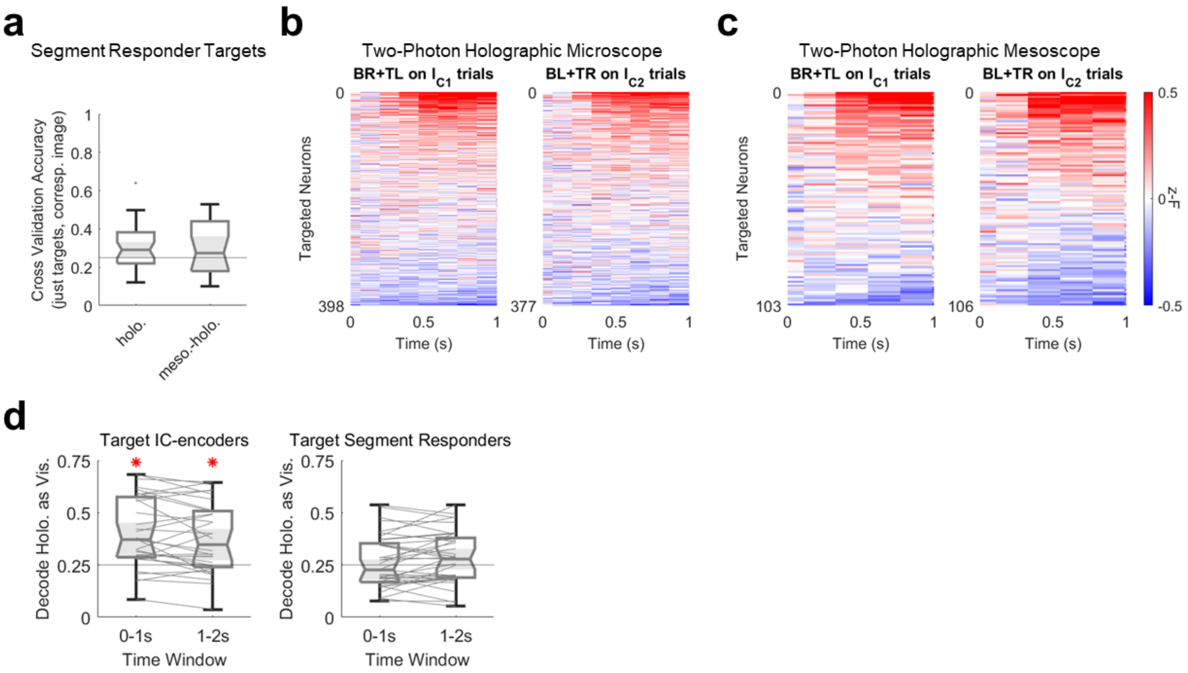

Supplementary Figure 6. V1 segment responders photoactivation results in 2p holographic microscope experiments (Fig. 5, V1 field-of-view) and 2p holographic mesoscope experiments (Fig. 6) are comparable.

a, Cross validation accuracy of visual responses for holographically targeted segment responder neurons on corresponding visual trials, for 2p holographic microscope vs 2p holographic mesoscope experiments. (n=37 vs n=22 ensembles with ≥10 targets; Wilcoxon rank sum test p=0.8754).

b, Visual responses of holographically targeted segment responder neurons in the 2p holographic microscope. Field-of-view was in V1 layer 2/3 in all experiments.

c, Visual responses of holographically targeted segment responder neurons in the 2p holographic mesoscope. All holographically targeted neurons were in V1 layer 2/3.

d, The holographic artifacts during photoactivation could be successfully removed in the holographic V1 dataset, but not in the holographic mesoscope dataset. As such, the time window analyzed in holographic V1 experiments (0 – 1s relative to holography onset, i.e., concurrent with photostimulation, Fig. 5) was different from holographic mesoscope experiments (1 – 2s relative to holography onset, i.e., time period immediately following photostimulation offset, Fig. 6). Comparison of these time windows in the holographic V1 dataset suggests that the effects found in the holographic mesoscope dataset were likely underestimated (n=24 sessions from 4 mice; *p<0.05 Wilcoxon signed-rank test compared to chance performance of 0.25; *Left* shows effects of photoactivating IC-encoders p=0.0001, 0.0009; *Right* shows effects of photoactivating segment responders p=0.2304, 0.6454). Box-and-whisker plots are formatted as follows: center line, median; box limits, upper and lower quartiles; whiskers, 1.5x interquartile range; circles, outliers. Gray lines indicate paired data for each session.

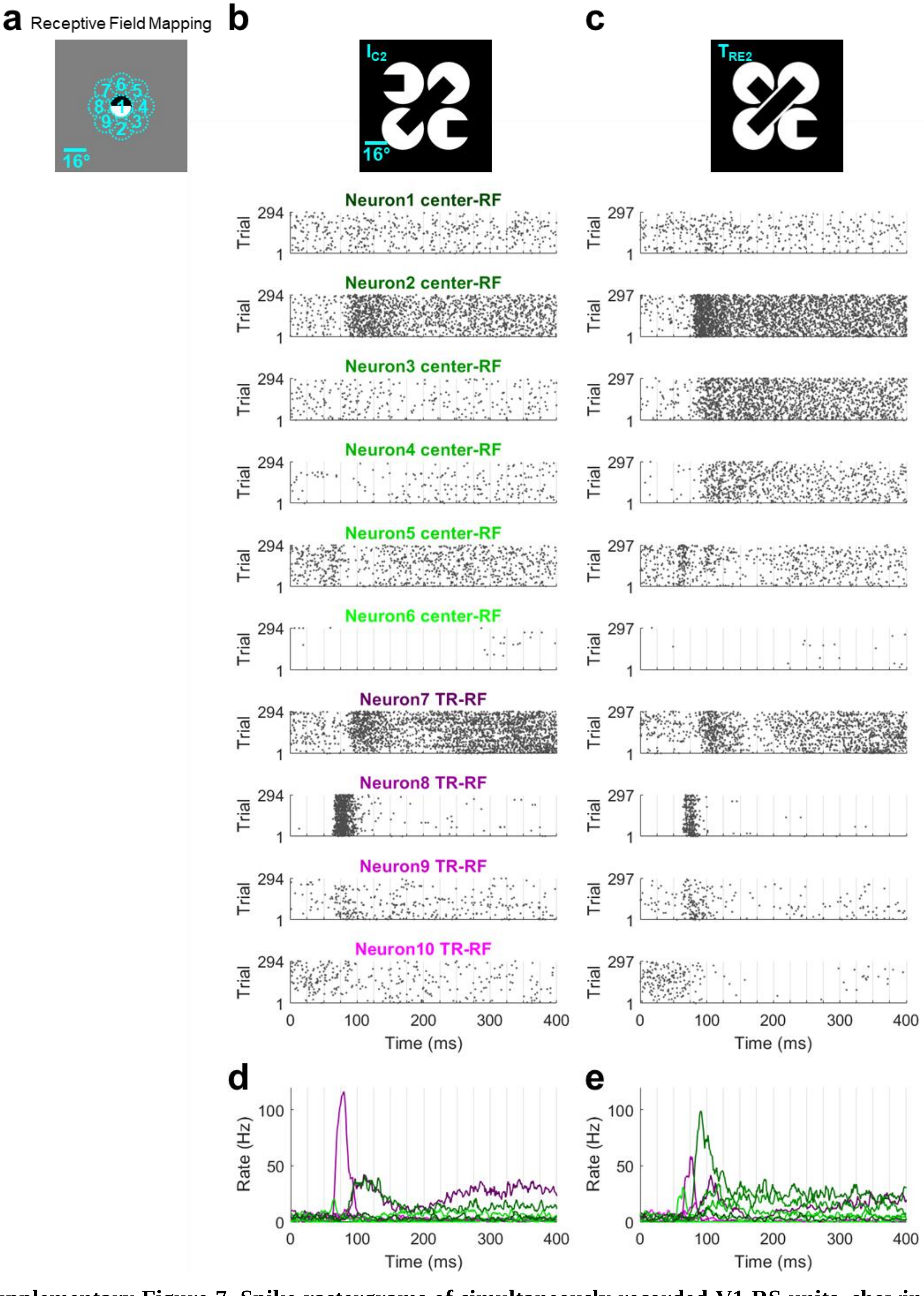

Supplementary Figure 7. Spike rastergrams of simultaneously recorded V1 RS units, showing that on I_C trials, neurons with receptive fields on segments do not systematically spike earlier than neurons with receptive fields on the illusory gap region.

a, Receptive fields were mapped with 16 visual degree diameter circular patches of drifting gratings appearing in 9 different positions.

b, Spike rastergrams on fixed-gaze I_{C2} presentation trials; mouse eye position stayed within 8 visual degrees of the mode eye position throughout the entire 400ms of image presentation. Six neurons with receptive fields on the illusory gap region (center-RF; position 1) and four neurons with receptive fields on the top-right segment (TR-RF; position 5) are shown. These neurons were simultaneously recorded in V1 with a Neuropixels probe.

c, Same as **b**, but for fixed-gaze T_{RE2} presentation trials.

d, I_{C2} trial-averaged responses of rastergrams depicted in **b**, with neurons color coded as in **b**.

e, T_{RE2} trial-averaged responses of rastergrams depicted in **c**, with neurons color coded as in **b**.

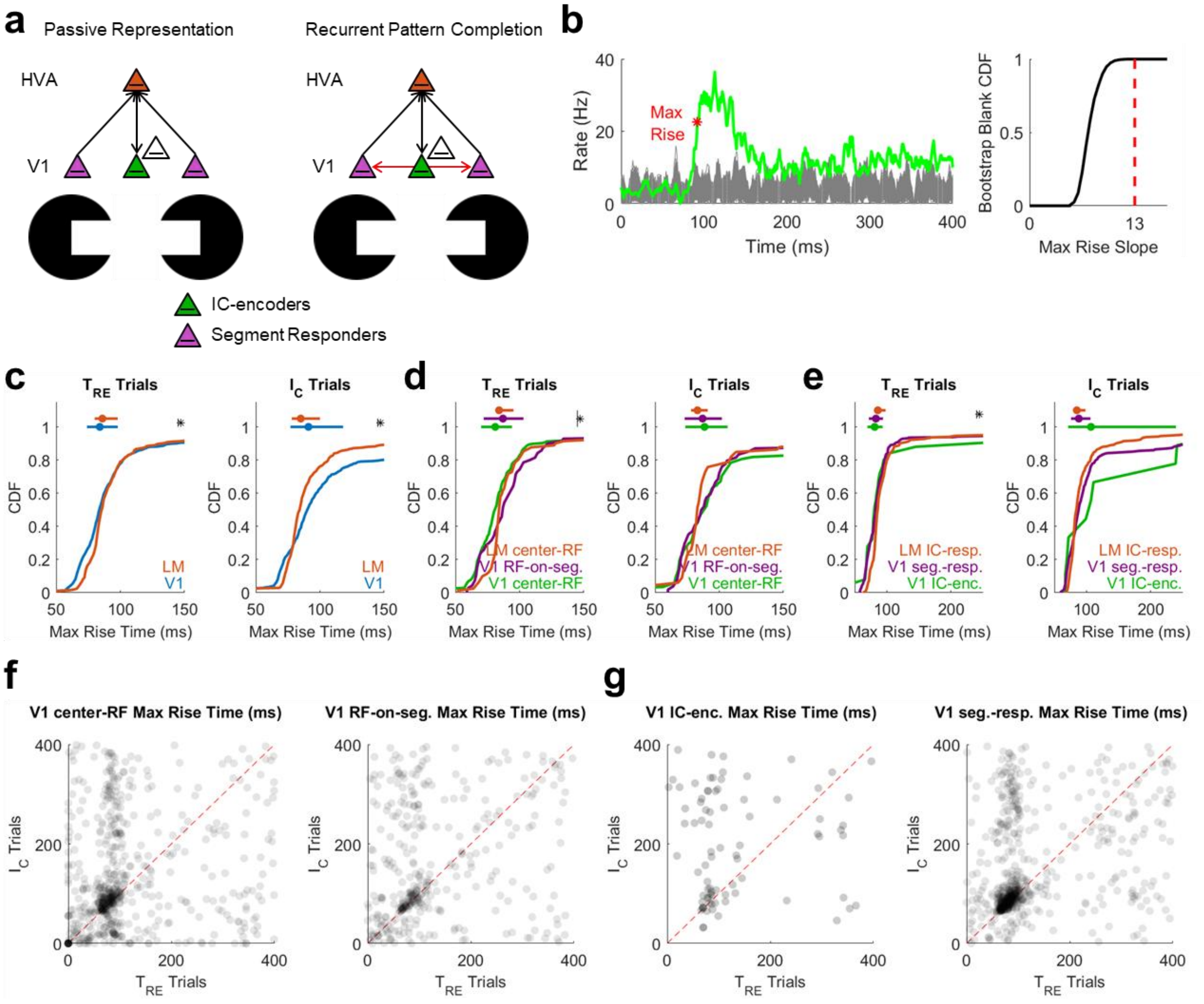

Supplementary Figure 8. Visual response latency data is inconsistent with the "passive representation" model of illusory contour encoding.

a, "Passive representation" model posits that V1 layer 2/3 passively represents illusory contour inference without contributing to encoding (*left*). Our proposed model, "recurrent pattern completion" model, posits that V1 layer 2/3 actively contributes to computing illusory contour inference via recurrent processing (*right*). The "passive representation" model predicts that V1 segment responders respond the same way to illusory and real bars, whereas the "recurrent pattern completion" model predicts that the responses of V1 segment responders are modulated by V1 IC-encoders.

b, For each neuron, max rise time was defined as the timepoint of the maximal PSTH increment in consecutive 5ms bins (max rise slope, Δrate/Δtime). Max rise time was considered valid only if max rise slope was higher than the 97.5 percentile of 1000X bootstrapped blank trials (gray traces in *left* panel).

c-g, Only valid max rise time were compared between groups of neurons.

c, Kruskal-Wallis test across RS units in V1 vs LM on T_{RE} trials (*left*, p=0.0032; V1 valid n=725, median 84ms, Q1 – Q3 74 – 98ms; LM valid n=416, median 86ms, Q1 – Q3 80 – 98ms), and I_C trials (*right*, p=0.0059; V1 valid n=383, median 91ms, Q1 – Q3 77 – 118ms; LM valid n=382, median 85ms, Q1 – Q3 78 – 100ms). *p<0.05.

d, Kruskal-Wallis test across V1 RS units with receptive fields in the center position between segments vs V1 RS units with receptive fields on the inducing segments (BR+TL for T_{RE1} / I_{C1} trials, BL+TR for

T_{RE2} / I_{C2} trials) vs LM RS units with receptive fields in the center position: T_{RE} trials to the *left* (p=0.0054; V1 center-RF valid n=205, median 81ms, Q1 – Q3 70 – 94ms; V1 RF-on-segments valid n=70, median 87ms, Q1 – Q3 72 – 103ms; LM center-RF valid n=81, median 84ms, Q1 – Q3 82 – 95ms) and IC trials to the *right* (p=0.8681; V1 center-RF valid n=88, median 89ms, Q1 – Q3 74 – 107ms; V1 RF-on-segments valid n=54, median 87ms, Q1 – Q3 73 – 102ms; LM center-RF valid n=66, median 83ms, Q1 – Q3 78 – 91ms), post-hoc Tukey-Kramer test *p<0.05.

e, Kruskal-Wallis test across RS units that are V1 IC-encoders vs V1 segment responders (for inducing segments; BR+TL for T_{RE1} / I_{C1} trials, BL+TR for T_{RE2} / I_{C2} trials) vs LM I_C-responsive neurons: T_{RE} trials to the *left* ($p=4.2\times10^{-5}$; V1 IC-encoders valid n=25, median 81ms, Q1 – Q3 70 – 94ms; V1 segment responders valid n=292, median 83ms, Q1 – Q3 72 – 94ms; LM I_C-responsive neurons valid n=332, median 86ms, Q1 – Q3 81 – 98ms) and IC trials to the *right* (p=0.4697; V1 IC-encoders valid n=9, median 107ms, Q1 – Q3 72 – 240ms; V1 segment responders valid n=221, median 88ms, Q1 – Q3 76 – 106ms; LM I_C-responsive neurons valid n=325, median 85ms, Q1 – Q3 79 – 99ms), post-hoc Tukey-Kramer test *p<0.05.

f, Scatter plot comparing max rise time on T_{RE} trials (x-axis) vs I_C trials (y-axis), for V1 RS units with receptive fields in the center position (*left*, n=620, Wilcoxon signed-rank test $p=4.6\times10^{-5}$), or on the inducing segments (*right*, n=356, p=0.0001).

g, Scatter plot comparing max rise time on T_{RE} trials (x-axis) vs I_C trials (y-axis), for V1 RS units that are IC-encoders (*left*, n=82, Wilcoxon signed-rank test p=0.0002), or segment responders (*right*, n=665, $p=1.2\times10^{-9}$).

Supplementary References

1 Lee, T. S. & Nguyen, M. Dynamics of subjective contour formation in the early visual cortex. *Proc Natl Acad Sci U S A* **98**, 1907-1911 (2001). https://doi.org/10.1073/pnas.98.4.1907

2 von der Heydt, R., Peterhans, E. & Baumgartner, G. Illusory contours and cortical neuron responses. *Science* **224**, 1260-1262 (1984). https://doi.org/10.1126/science.6539501

3 Lyall, E. H. *et al.* Synthesis of a comprehensive population code for contextual features in the awake sensory cortex. *Elife* **10** (2021). https://doi.org/10.7554/eLife.62687

4 Fu, J. *et al.* Pattern completion and disruption characterize contextual modulation in the visual cortex. *bioRxiv* (2024). https://doi.org/10.1101/2023.03.13.532473

5 Keller, A. J., Roth, M. M. & Scanziani, M. Feedback generates a second receptive field in neurons of the visual cortex. *Nature* **582**, 545-549 (2020). https://doi.org/10.1038/s41586-020-2319-4

6 Young, H., Belbut, B., Baeta, M. & Petreanu, L. Laminar-specific cortico-cortical loops in mouse visual cortex. *Elife* **10** (2021). https://doi.org/10.7554/eLife.59551

7 Siu, C., Balsor, J., Merlin, S., Federer, F. & Angelucci, A. A direct interareal feedback-to-feedforward circuit in primate visual cortex. *Nat Commun* **12**, 4911 (2021). https://doi.org/10.1038/s41467-021-24928-6

8 Lee, T. S. Computations in the early visual cortex. *J Physiol Paris* **97**, 121-139 (2003). https://doi.org/10.1016/j.jphysparis.2003.09.015

9 Wyatte, D., Jilk, D. J. & O'Reilly, R. C. Early recurrent feedback facilitates visual object recognition under challenging conditions. *Front Psychol* **5**, 674 (2014). https://doi.org/10.3389/fpsyg.2014.00674

10 Das, A. & Fiete, I. R. Systematic errors in connectivity inferred from activity in strongly recurrent networks. *Nat Neurosci* **23**, 1286-1296 (2020). https://doi.org/10.1038/s41593-020-0699-2

11 Keller, D., Ero, C. & Markram, H. Cell Densities in the Mouse Brain: A Systematic Review. *Front Neuroanat* **12**, 83 (2018). https://doi.org/10.3389/fnana.2018.00083
