# Supplementary material for: Recurrent pattern completion drives the neocortical representation of sensory inference
Source: Nat Neurosci. 2025 Sep 15;28(11):2319–29. doi: 10.1038/s41593-025-02055-5 (PMC12586158; doi:10.1038/s41593-025-02055-5)
Supplement: Supplementary file 2 — Reporting Summary [file 41593_2025_2055_MOESM2_ESM.pdf]

nature portfolio

Corresponding author(s): Hillel Adesnik

Last updated by author(s): March 25, 2025

Reporting Summary

Nature Portfolio wishes to improve the reproducibility of the work that we publish. This form provides structure for consistency and transparency in reporting. For further information on Nature Portfolio policies, see our Editorial Policies and the Editorial Policy Checklist.

Statistics

For all statistical analyses, confirm that the following items are present in the figure legend, table legend, main text, or Methods section.

n/a	Confirmed	
☐	☒	The exact sample size (n) for each experimental group/condition, given as a discrete number and unit of measurement
☐	☒	A statement on whether measurements were taken from distinct samples or whether the same sample was measured repeatedly
☐	☒	The statistical test(s) used AND whether they are one- or two-sided<br>*Only common tests should be described solely by name; describe more complex techniques in the Methods section.*
☐	☒	A description of all covariates tested
☐	☒	A description of any assumptions or corrections, such as tests of normality and adjustment for multiple comparisons
☐	☒	A full description of the statistical parameters including central tendency (e.g. means) or other basic estimates (e.g. regression coefficient) AND variation (e.g. standard deviation) or associated estimates of uncertainty (e.g. confidence intervals)
☐	☒	For null hypothesis testing, the test statistic (e.g. F, t, r) with confidence intervals, effect sizes, degrees of freedom and P value noted<br>*Give P values as exact values whenever suitable.*
☒	☐	For Bayesian analysis, information on the choice of priors and Markov chain Monte Carlo settings
☒	☐	For hierarchical and complex designs, identification of the appropriate level for tests and full reporting of outcomes
☐	☒	Estimates of effect sizes (e.g. Cohen's d, Pearson's r), indicating how they were calculated

Our web collection on statistics for biologists contains articles on many of the points above.

Software and code

Policy information about availability of computer code

Data collection	Scanimage v2018b, Scanbox, OpenEphys GUI (https://github.com/open-ephys/plugin-GUI)
Data analysis	Suite2p, Kilosort2<br>Custom analysis code is published in Code Ocean: https://codeocean.com/capsule/6659254/tree

For manuscripts utilizing custom algorithms or software that are central to the research but not yet described in published literature, software must be made available to editors and reviewers. We strongly encourage code deposition in a community repository (e.g. GitHub). See the Nature Portfolio guidelines for submitting code & software for further information.

Data

Policy information about availability of data

All manuscripts must include a data availability statement. This statement should provide the following information, where applicable:

- Accession codes, unique identifiers, or web links for publicly available datasets
- A description of any restrictions on data availability
- For clinical datasets or third party data, please ensure that the statement adheres to our policy

Neuropixels dataset is available at: https://dandiarchive.org/dandiset/000248/ (See also https://openscopedatafront.web.app/table)
Postprocessed data is available at https://codeocean.com/capsule/6659254/tree.

Research involving human participants, their data, or biological material

Policy information about studies with human participants or human data. See also policy information about sex, gender (identity/presentation), and sexual orientation and race, ethnicity and racism.

Reporting on sex and gender	N/A
Reporting on race, ethnicity, or other socially relevant groupings	N/A
Population characteristics	N/A
Recruitment	N/A
Ethics oversight	N/A

Note that full information on the approval of the study protocol must also be provided in the manuscript.

Field-specific reporting

Please select the one below that is the best fit for your research. If you are not sure, read the appropriate sections before making your selection.

☒ Life sciences ☐ Behavioural & social sciences ☐ Ecological, evolutionary & environmental sciences

For a reference copy of the document with all sections, see nature.com/documents/nr-reporting-summary-flat.pdf

Life sciences study design

All studies must disclose on these points even when the disclosure is negative.

Sample size	We did not use a statistical method to predetermine sample size but we used equivalent or larger sample size than similar studies. When applicable, we used cross-validation to minimize reliance on a critical number.
Data exclusions	All data exclusions are described along with exclusion criteria and the rationale for exclusion.<br>In the Neuropixels dataset, for each visual cortical area, sessions with <50 RS units in that area are excluded. On the day of the recording, six Neuropixels probes were targeted to their respective visual cortical areas. Probe locations were confirmed post-hoc ex-vivo and registered to the Allen Brain Common Coordinate Framework. We used 50 RS units as a criteria to determine whether probe targeting was successful.<br>In the mesoscope two-photon imaging dataset, for each visual cortical area, sessions with <100 neurons in that area are excluded. We mapped visual areas via retinotopy. When the window quality was suboptimal, some of the smaller higher visual areas were not identifiable. We used 100 neurons as a criteria to determine whether visual area identification was successful.<br>In the two-photon holographic optogenetics and two-photon holographic mesoscope experiments, for each holographic ensemble, sessions with <10 targets were excluded. During online analysis, we identified neurons in each functional group (IC1-encoders, IC2-encoders, BR/TL/BL/TR-segment responders). Due to the sparseness of IC-encoders, there were sessions with <10 IC-encoder ensembles. Because the mode receptive field of the two-photon imaging field-of-view was aligned to the center of the monitor, there were sessions with <10 segment responder ensembles. Because the network effects of photoactivation are small and noisy when the holographic ensembles are small, we excluded holographic ensembles with <10 targets.
Replication	This paper includes datasets collected across 4 different experimental setups (1 Neuropixels recording setup at the Allen Institute and 3 two-photon microscopes, including a mesoscope, in the Adesnik Lab at UC Berkeley; see Methods for details). All datasets have been internally replicated. In the Neuropixels dataset, one session was recorded per mouse (12 sessions from 12 mice). In two-photon experiments, several sessions may have been recorded from one mouse, but we confirmed the session-level effects at the mouse-level (29 sessions from 5 mice for V1 layer 2/3 imaging dataset in Figure 1; 11 sessions from 5 mice for V1 layer 2/3 imaging dataset in Figure 3; 8 sessions from 2 mice for V1 layer 4 imaging dataset in Figure 3; 19 sessions from 5 mice for mesoscope imaging dataset in Figure 3; 24 sessions from 4 mice for two-photon holographic optogenetics experiments in Figures 4-5; 11 sessions from 2 mice for two-photon holographic mesoscope experiments in Figure 6).<br>In addition, our main conclusions have been successfully replicated across datasets. Analysis of neural activity during visual presentation of illusory contours and related images have been replicated between Neuropixels and two-photon imaging, and across two-photon imaging datasets collected in different microscopes, imaging conditions, transgenic mouse lines and GCaMP variations (GCaMP6s, GCaMP6m and GCaMP7s).<br>Selective two-photon holographic optogenetics experiments have been replicated across two distinct microscopes; a standard 3D-SHOT two-photon microscope and a two-photon holographic mesoscope. In addition, we used two different opsins; ChRmine and ChROME. Despite these variations, we confirmed that the experimental conditions are comparable and that the results are consistent.<br>Overall, we found excellent replication and reproducibility of our results, both within and across datasets.
Randomization	Trial order was randomized in all experiments.
Blinding	Blinding is not applicable to our study. We targeted specific brain areas for recording, but beyond this, we did not pre-select neurons to record. As such, our results are representative of all recordings from that specific brain area.

Reporting for specific materials, systems and methods

We require information from authors about some types of materials, experimental systems and methods used in many studies. Here, indicate whether each material, system or method listed is relevant to your study. If you are not sure if a list item applies to your research, read the appropriate section before selecting a response.

Materials & experimental systems

n/a	Involved in the study
☒	☐ Antibodies
☒	☐ Eukaryotic cell lines
☒	☐ Palaeontology and archaeology
☐	☒ Animals and other organisms
☒	☐ Clinical data
☒	☐ Dual use research of concern
☒	☐ Plants

Methods

n/a	Involved in the study
☒	☐ ChIP-seq
☒	☐ Flow cytometry
☒	☐ MRI-based neuroimaging

Animals and other research organisms

Policy information about studies involving animals; ARRIVE guidelines recommended for reporting animal research, and Sex and Gender in Research

Laboratory animals	All experiments were performed in mice of both sexes, aged 6 weeks and older. Imaging experiments in Fig. 1-3 were conducted with CaMKII-tTA;tetO-GcaMP6s mice (V1 layer 2/3 and Mesoscope layer 2/3 data), and Scnn1a-Tg3-Cre;Ai162(TIT2L-GC6s-ICL-tTA2)-D mice (V1 layer 4 data). Neuropixels experiments were conducted with Sst-IRES-Cre;Ai32(RCL-ChR2(H134R)_EYFP) mice (n=10) and PV-IRES-Cre;Ai32(RCL-ChR2(H134R)_EYFP) mice (n=4). 2p holographic optogenetics experiments in Fig. 4-5 were conducted with AAV8-hSyn-GCaMP6m-p2A-ChRmine-Kv2.1-WPRE injected in PV-IRES-Cre;RCL-tdTomato mice (n=2) and AAV9-2YF-hSyn-DIO-GcaMP6m-P2A-ChRmine-Kv2.1-WPRE in Emx1-Cre mice (n=2). 2p holographic mesoscope experiments in Fig. 6 were conducted with Vglut1-Cre;Ai203 mice (Ai203 refers to a transgenic line expressing the transgene TITL-st-ChroME-GCaMP7s-ICL-nls-mRuby3-IRES2-tTA2; n=2).<br>All mice were housed and maintained on a reverse 12-hour light cycle in a shared facility with room temperatures between 68 and 73 °F, and humidity between 30 and 70 %.
Wild animals	No wild animals were used in this study.
Reporting on sex	Mice of both sexes were used.
Field-collected samples	No field-collected samples were used in this study.
Ethics oversight	All experiments on animals were conducted with approval of the Animal Care and Use Committee of the University of California, Berkeley (2p experiments) and Allen Institute's Institutional Animal Care and Use Committee (Neuropixels experiments).

Note that full information on the approval of the study protocol must also be provided in the manuscript.

Plants

Seed stocks	*Report on the source of all seed stocks or other plant material used. If applicable, state the seed stock centre and catalogue number. If plant specimens were collected from the field, describe the collection location, date and sampling procedures.*
Novel plant genotypes	*Describe the methods by which all novel plant genotypes were produced. This includes those generated by transgenic approaches, gene editing, chemical/radiation-based mutagenesis and hybridization. For transgenic lines, describe the transformation method, the number of independent lines analyzed and the generation upon which experiments were performed. For gene-edited lines, describe the editor used, the endogenous sequence targeted for editing, the targeting guide RNA sequence (if applicable) and how the editor was applied.*
Authentication	*Describe any authentication procedures for each seed stock used or novel genotype generated. Describe any experiments used to assess the effect of a mutation and, where applicable, how potential secondary effects (e.g. second site T-DNA insertions, mosiacism, off-target gene editing) were examined.*
